# Hypoxia promotes metastasis by relieving miR-598-3p-restricted glycolysis in gastric cancer

**DOI:** 10.1186/s12967-024-04957-7

**Published:** 2024-03-15

**Authors:** Wei Zhou, Mengyuan Tang, Dan He, Yi Shen, Ziwei Huang, Wenxin Xia, Zhiyun Wu, Wenxiang Wei, Hui Zheng, Qi Wang, Weifeng Shi, Jingting Jiang

**Affiliations:** 1 Department of Clinical Laboratory, The Third Affiliated Hospital of Soochow University, ChangZhou, 213003 Jiangsu China; 2 Department of Immunology, Soochow University, SuZhou, 215004 Jiangsu China; 3 Department of Cell Biology, Soochow University, SuZhou, 215004 Jiangsu China; 4 Institutes of Biology and Medical Science (IBMS), Soochow University, SuZhou, 215004 Jiangsu China; 5 Department of Biological Treatment, The Third Affiliated Hospital of Soochow University, ChangZhou, 213003 Jiangsu China

**Keywords:** Glucose metabolism, Metastasis, Hypoxia, microRNAs, Gastric cancer

## Abstract

The activation of glycolysis, particularly in the context of reprogrammed energy metabolism, is increasingly recognized as a significant characteristic of cancer. However, the precise mechanisms by which glycolysis is promoted in metastatic gastric cancer cells under normal oxygen conditions remain poorly understood. MicroRNAs (miRNAs) play a crucial role in the development of malignant phenotypes in gastric cancer. Nevertheless, our understanding of the specific involvement of miRNAs in hypoxia-induced metabolic shifting and the subsequent metastatic processes is limited. Hypoxia-induced downregulation of miR-598-3p mechanistically leads to the upregulation of RMP and IGF1r, thereby promoting glycolysis. Either overexpression of miR-598-3p or R406 treatment effectively suppresses the metastasis of gastric cancer cells both in vitro and in vivo. Collectively, the depletion of miR-598-3p alters glucose metabolism from oxidative phosphorylation to glycolysis, thereby exacerbating the malignancy of gastric cancer cells. The present findings indicate a potential target for the development of therapeutics against gastric cancers with increased miR-598-3p expression.

**Supplementary Information:**

The online version contains supplementary material available at 10.1186/s12967-024-04957-7.

## Introduction

Gastric cancer and other solid tumors have the common characteristics of fast growth and metastasis. However, the rapid growth of gastric cancer often outpaces the development of functional blood vessels, and there is frequently insufficient O_2_ supplementation in the regions of cancer [1–3]. Therefore, tumor cells exist in a hypoxic microenvironment, which is a fundamental solid tumor microenvironment feature. The ability of cancer cell to adjust their glucose metabolism from oxidative phosphorylation to glycolysis is in response to the hypoxic microenvironment, a metabolic adaptation that confers significant benefits such as enhanced energy production and heightened activity of intermediates in various metabolic pathways. The continuous production of acidic lactate alters the tumor microenvironment, therefore impairing tumor immune responses and ultimately leading to tumor progression [4–6]. Although the essential role of glycolysis in solid tumors has been widely investigated, the roles of glycolysis in the metastasis of hypoxic gastric cancer cells remain poorly understood. Accordingly, clarification of this mechanism is necessary for understanding the connection between glucose metabolism shifting and metastasis driven by hypoxia.

In hypoxia, the expression of the glycolytic genes PKM2 (pyruvate kinase M2) [7], HK2 (hexokinase 2), and LDHA (lactate dehydrogenase A) [8, 9] is upregulated, which promotes glycolysis in tumor cells. On the other hand, some oncogenes cooperate with glycolytic genes, subsequently promoting the metastasis of hypoxic tumor cells [9, 10]. Therefore, uncovering the underlying mechanism between metastasis and glycolysis in hypoxic gastric cancer cells is worthwhile. The insufficiency of members within the tumor suppressor miRNA biogenesis pathway highlights the pro-carcinogenic impact of decreased oxygen levels within tumors. Recent findings have shown that hypoxia induces a global reduction in miRNA expression in ovarian and breast cancer, thereby promoting epithelial-mesenchymal transition (EMT), which encompasses dynamic changes in cellular organization from epithelial to mesenchymal phenotypes, finally leads to functional changes in cell migration and invasion. This suggests a connection between the EMT-promoting effect of hypoxia and the deregulation of miRNA in these tumors. However, due to the tumor-specific and manifold influence of hypoxia and the divergence of miRNAs and vice versa, the crosstalk between hypoxic signaling and miRNA biogenesis is still relatively unexplored, and the potential therapeutic setting remains to be established. Multiple studies have demonstrated that the miR-598 family functions as a tumor suppressor and exhibits downregulation in various types of cancer. Specifically, miR-598-3p (3ʹ end transcript of miR-598) was downregulated in triple-negative breast cancer [11], retinoblastoma [12] and colorectal cancer [13], miR-598-5p was downregulated in osteosarcoma [14] and gastric cancer [15]. Both miR-598-3p and -5p was shown to suppress the proliferation and metastasis of multiple cancers and was associated with poor prognosis. However, the molecular mechanism by which miR-598-3p was not well studied in the regulation of glucose metabolism and the consequent metastasis of gastric cancer cells during hypoxia. Based on this, we screened candidate miRNAs that may be involved in hypoxic gastric cancer cell, and we reported that loss of miR-598-3p could promotes metastasis through facilitating glycolysis in gastric cancer cells. Additionally, miR-598-3p can directly targets RMP and IGF1r, the key regulators of glucose metabolism and relevant malignant phenotypes of cancer cells.

## Material and methods

### Patients and tissue specimens

A total of 20 gastric cancer patients were recruited in the current study. All patients underwent curative resection for gastric cancer at the Third Affiliated Hospital of Soochow university (Changzhou, Jiangsu Province, China). Prior to the surgery, no patients received any local or systemic anticancer treatments. This study was approved by the Ethics Committee of Third Affiliated Hospital of Soochow University. The informed content are listed in Additional file 3.

### In vivo pulmonary and peritoneal metastasis assay

Female BALB/c nude mice (6–8 weeks old) were purchased from the animal experiment center of Soochow University and maintained and treated under specific pathogen-free conditions. All animal protocols were approved by the Institutional Laboratory Animal Care and Use Committee at Soochow University. For the pulmonary metastasis assay, mice were injected intravenously with 2 × 10^6^ gastric cancer cells or PBS via the tail vein. For the peritoneal metastasis assay, mice were injected peritoneally with 5 × 10^6^ gastric cancer cells or PBS. If necessary, 10 mg/kg/day UK5099 (Selleck, S5317, C18H12N2O2) or 1 mg/kg/day R406 (Selleck, S1533, C22H23FN6O5) was peritoneally injected after 2 weeks. Mice were killed via cervical dislocation, and the lungs and peritoneal cavity were photographed at 4 weeks. The survival of mice was recorded, and a Kaplan‒Meier survival curve was generated.

### RNA extraction and quantitative real-time PCR analysis

Total RNA was isolated from cultured cells or tissue using TRIzol (Thermo Fisher Scientific). The RNA then underwent cDNA synthesis using the RevertAid First Strand cDNA Synthesis Kit (Thermo Fisher Scientific). Quantitative real-time polymerase chain reaction (qPCR) was performed using Power SYBR green master mix (Thermo Fisher Scientific) or TaqMan Universal Master Mix (Thermo Fisher Scientific). The results were normalized to the internal controls GAPDH or RUN44. Predesigned primer and probe set for testing the miR-598-3p (A25576), miR-210 (4427975), miR-1281 (4427975), miR-3162-3p (4427975), miR-197-3p (4427975), miR-4649-3p (4440886) and control RUN44 (4427975) genes were used (Thermo Fisher Scientific). The primers used for qPCR are listed in Additional file 1.

### Western blotting

Total cell lysates were obtained by M-PER™ Mammalian Protein Extraction Reagent (Thermo Fisher Scientific) with Halt Protease and Phosphatase Inhibitor (Thermo Fisher Scientific). Protein samples were separated by sodium dodecyl sulfate‒polyacrylamide gel electrophoresis (SDS‒PAGE) and then transferred onto polyvinylidene fluoride membranes (Millipore). The membranes were blocked in TBST with 5% BSA and then incubated overnight at 4 °C with primary antibody. The dilution of each antibody was based on manual instructions. The membranes were washed 4 times for 5 min with TBST and incubated for 60 min with secondary antibody. After 4 further washes, bands were detected using enhanced chemiluminescence (Millipore). Images were captured by Gbox Chemi-XR 5 (Syegene) and quantitated using Quantity One (Bio-Rad). The antibodies are listed in Additional file 2.

### Measurement of glucose consumption and lactate production

To detect glucose consumption and lactate production, 2 × 10^5^ BGC-823 or 1 × 10^5^ MKN45 cells were plated and incubated in culture medium with 15 mM glucose in the absence of glutamine and pyruvate for 5 h. After washing with PBS, the culture medium and cells were harvested separately. The concentrations of glucose and lactate in the culture medium were measured using a glucose assay and lactate assay kit (Solarbio Life Science, Beijing, China), respectively, according to the manufacturer’s instructions.

### Measurement of ATP level

ATP levels in GC cells were measured using an ATP assay kit (Solarbio Life Science, Beijing, China) according to the manufacturer’s instructions. To measure the levels of glycolytic or mitochondrial ATP production, 2 × 10^5^ BGC-823 or 1 × 10^5^ MKN45 cells were seeded into plates and incubated in media containing 100 nM oligomycin A (Selleck) or 10 mM pyruvate in the absence of glucose and glutamine for 5 h. After being washed with PBS, the cells were lysed and examined for ATP levels using an ATP assay kit according to the manufacturer’s protocol.

### Statistical analysis

Statistical analysis was performed with SPSS software and GraphPad Prism 9 software. Potential differences were analyzed using Student’s t test with or without Welch’s correction between two groups and one-way or two-way ANOVA with multiple testing corrections within multiple groups. Data are expressed as the mean ± SD.* p* < 0.05 was considered significant. The material and methods of  Cell culture, transfection, and virus; Analysis of miRNA Chip and Transcriptome Sequencing; Dual-luciferase reporter assay; Cell invasion assay and Wound healing assay are offered in Additional file 4.

## Results

### Integrated analysis of key miRNAs involved in hypoxic gastric cancer cells

**Fig. 1:**
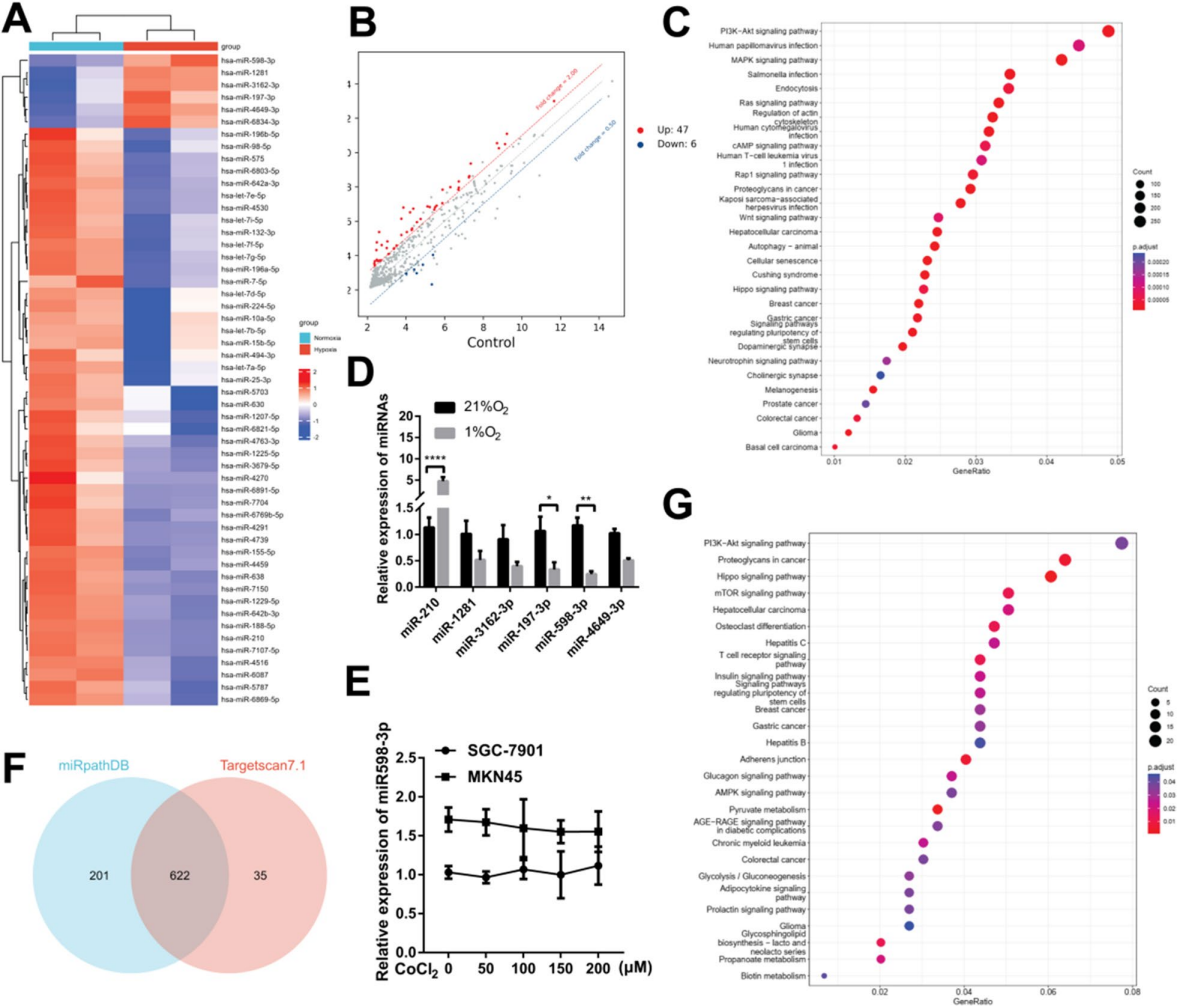
Integrated analysis of key miRNAs involved in hypoxic gastric cancer cells. **A** Heatmap of differentially expressed miRNAs of miRNA Chip of MKN45 cells cultured in 21% O_2_ (normoxia) or 1% O_2_ (hypoxia). (Fold change ≥ 2, p value < 0.01). **B** Scatter plot showing the upregulated and downregulated miRNAs induced by hypoxia based on miRNA Chip analysis (Fold change ≥ 2, p value < 0.01). **C** Kyoto Encyclopedia of Genes and Genomes (KEGG) pathway enrichment analysis of the miRNAs identified to be differentially expressed between the normoxia and hypoxia groups (P < 0.05 by Fisher’s exact test). **D** Real-time PCR analysis of the indicated miRNA levels in SGC-7901 cells cultured in 21%O_2_ or 1%O_2_. **E** Real-time PCR analysis of miR-598-3p levels in SGC-7901 or MKN45 cells treated with the indicated concentrations of CoCl_2_. **F** Venn diagram showing prognostic genes derived from online tools of miRpathDB (https://mpd.bioinf.uni-sb.de/) and Targetscan 7.1 (https://www.targetscan.org/vert_71/). **G** KEGG pathway enrichment analysis of the miRNAs identified to be differentially expressed between the normoxia and hypoxia groups (P < 0.05 by Fisher’s exact test)

To determine the miRNAs that exhibited significant changes in hypoxic gastric cancer cells, MKN45 cells were cultured under normoxic and hypoxic conditions (1% O_2_) and subjected to miRNA Chip analysis. A comparison of the differentially expressed miRNAs revealed that 47 miRNAs were upregulated, and 6 miRNAs were downregulated in normoxic vs hypoxic MKN45 (Fig. 1A, B). Recent findings have shown that hypoxia induces a global reduction of miRNA encompasses dynamic changes in cellular organization from epithelial to mesenchymal phenotypes, which leads to functional changes in cell migration and invasion. The accuracy of the microchip data was validated through quantitative real-time PCR analysis of the top 5 downregulated miRNAs miR-1281, -3162-3p, -197-3p, -598-3p, -4649-3p and hypoxia marker miR-210. Notably, miR-598-3p was found to be significantly downregulated in response to hypoxia (Fig. 1D). The target genes of total 53 differentially expressed miRNAs were predicted by miRWalk3.0 online tools [16], resulting in the identification of 14383 candidate target genes. Subsequently, the target genes underwent KEGG enrichment analysis, which revealed their significant association with the PI3K-Akt, MAPK, Hippo, and Wnt signaling pathways (Fig. 1C). Drawing from the hypoxia treatment, we hypothesize that HIF1α may play a role in regulating miR-598-3p expression in GC cells, thereby causing its downregulation. To assess the validity of this hypothesis, we cultured GC cells in escalating concentrations of CoCl_2_. The presence of CoCl_2_ notably hindered the degradation of HIF1α in GC cells. However, it did not result in a decrease in the expression of miR-598-3p (Fig. 1E), indicates the downregulation of miR-598-3p is not dependent on the accumulation of HIF1α. Next, we predicted targets of miR-598-3p by using miRpathDB and Targetscan 7.1 online tools [17], Venn diagram shows 622 targets genes (Fig. 1F). KEGG enrichment analysis of 622 genes were depicted in Fig. 1G, which revealed their significant association with Insulin, Pyruvate metabolism and Glycolysis signaling pathways.

### miR-598-3p suppressed hypoxia-induced metastasis in vitro and in vivo

**Fig. 2:**
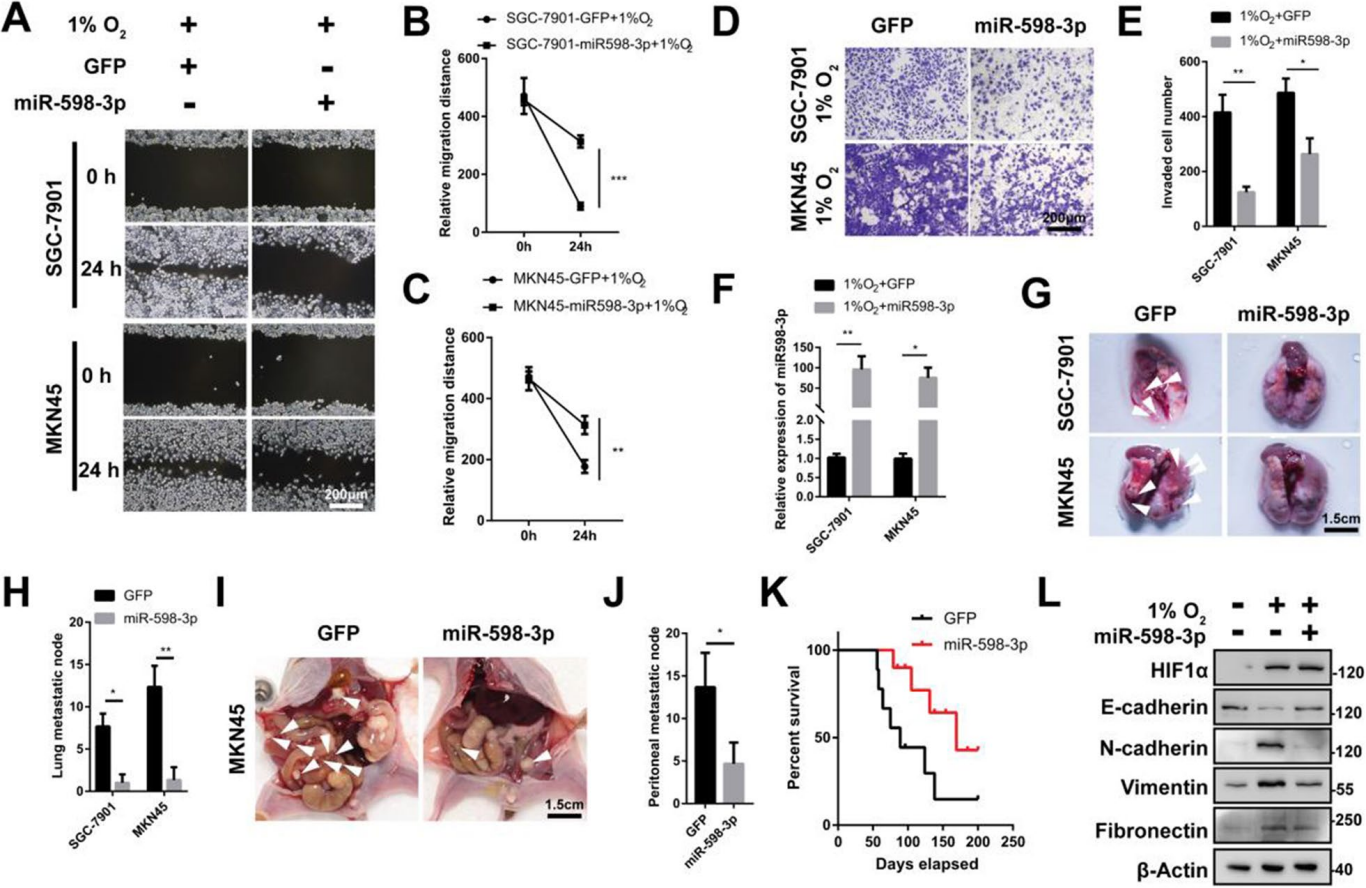
miR-598-3p suppressed hypoxia-induced metastasis in vitro and in vivo. **A**–**C** Wound-healing assay of the indicated cells transfected with GFP or miR-598-3p in 1%O_2_. **D**, **E** Transwell assay of SGC-7901 and MKN45 cells transfected with GFP or miR-598-3p in hypoxia (1%O_2_). **F** Real-time PCR analysis of miR-598-3p in SGC-7901 and MKN45 cells transfected with GFP or miR-598-3p. **G**, **H** BALB/c nude mice injected via the tail vein with SGC-7901 and MKN45 cells expressing GFP or miR-598-3p, respectively. The mice were sacrificed after 4 weeks, and the white arrow depicts metastatic nodes, n ≥ 5. **I**, **J** BALB/c nude mice intraperitoneally injected with MKN45 cells expressing GFP or miR-598-3p. The mice were sacrificed after 4 weeks, and the white arrow depicts a metastatic node, n ≥ 5. **K** Kaplan‒Meier survival curve of BALB/c nude mice injected via the tail vein with SGC-7901 cells expressing GFP or miR-598-3p, n = 10. **L** Western blot analysis of SGC-7901 cells transfected with miR-598-3p under hypoxia (1%O_2_)

There are several evidence of miR-598 family involved in cancer development, up-regulation of miR-598 attenuated the malignant phenotype of gastric cancer cells [15]. Consistent with previous finding, the overexpression of miR-598-3p in hypoxic SGC-7901 and MKN45 cells resulted in a significant decrease in cell migration (Fig. 2A–C, F). Furthermore, the overexpression of miR-598-3p also abolished hypoxia-induced cell invasion (Fig. 2D, E). The results of the pulmonary metastasis assay indicated a significant inhibition of metastatic node formation with the expression of miR-598-3p (Fig. 2G, H). To establish a peritoneal metastasis mouse model, MKN45 cells expressing GFP or miR-598-3p were intraperitoneally injected. Consistently, the expression of miR-598-3p resulted in suppression of metastatic nodes in the mesentery (Fig. 2I, J). Furthermore, miR-598-3p expression was found to prolong the survival of nude mice with pulmonary metastasis (Fig. 2K). The restoration of epithelial markers by miR-598-3p in a hypoxic environment resulted in a significant decrease in the expression of mesenchymal markers. Our in vitro and in vivo observations provide evidence that miR-598-3p plays a crucial role in the metastasis of gastric cancer.

### Silencing endogenous miR-598-3p promotes the metastasis of gastric cancer cells in vitro and in vivo

**Fig. 3:**
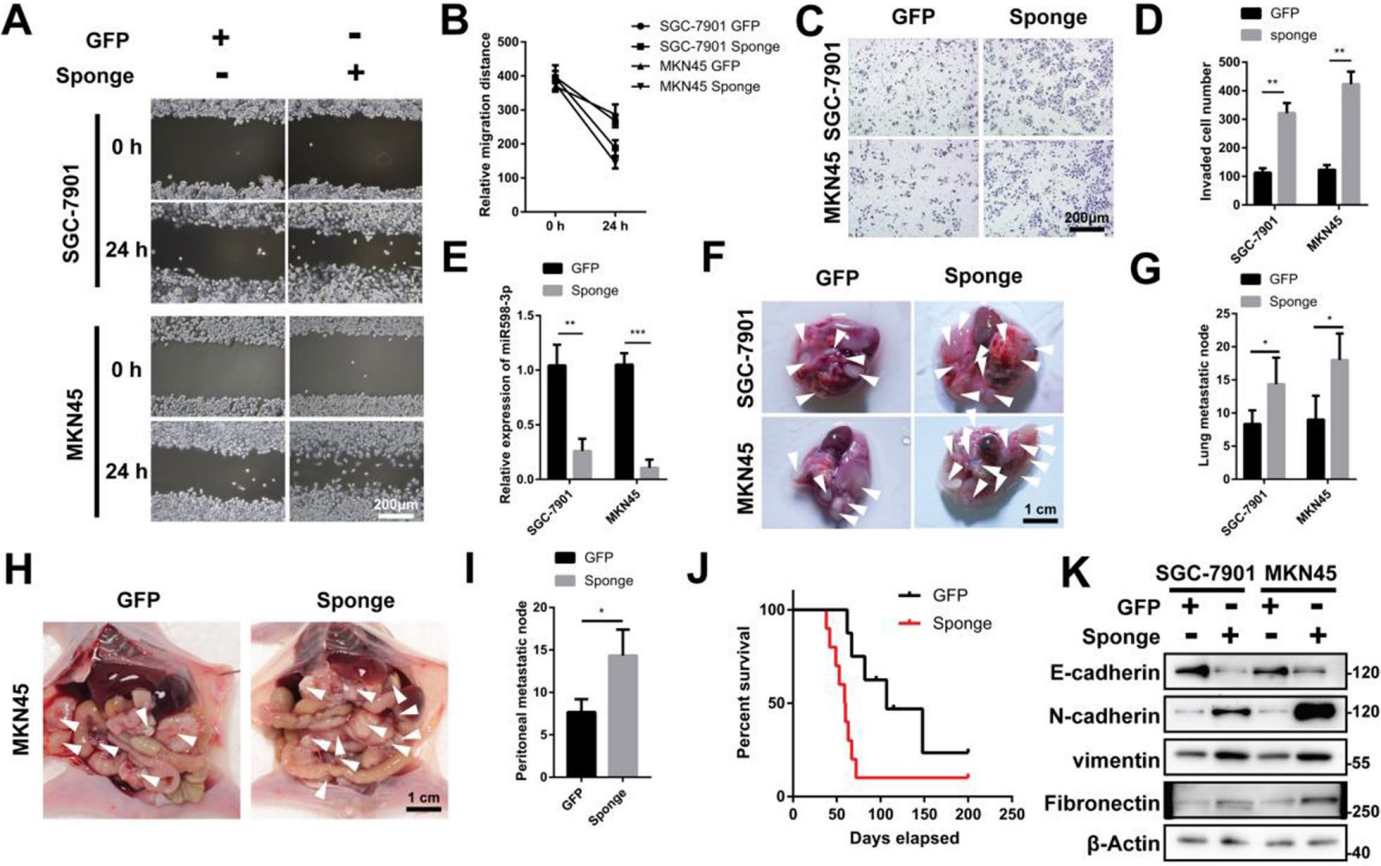
Silencing endogenous miR-598-3p promotes the metastasis of gastric cancer cells in vitro and in vivo. **A**, **B** Wound-healing assay of SGC-7901 and MKN45 cells transfected with GFP or the sponge of miR-598-3p. **C**, **D** Transwell assay of SGC-7901 and MKN45 cells transfected with GFP or the sponge of miR-598-3p. **E** Real-time PCR analysis of miR-598-3p levels in SGC-7901 and MKN45 cells transfected with GFP or the sponge of miR-598-3p. **F**, **G** BALB/c nude mice were injected via the tail vein with SGC-7901 and MKN45 cells transfected with GFP or the sponge of miR-598-3p. The white arrow depicts a metastatic node, n ≥ 5. **H**, **I** BALB/c nude mice injected intraperitoneally with MKN45 cells transfected with GFP or the sponge of miR-598-3p. The white arrow depicts a metastatic node, n ≥ 5. **J** Kaplan‒Meier survival curve of BALB/c nude mice injected via the tail vein with SGC-7901 cells transfected with GFP or the sponge of miR-598-3p, n = 10. **K** Western blot analysis of SGC-7901 and MKN45 cells transfected with GFP or the sponge of miR-598-3p

To further investigate whether endogenous miR-598-3p contributed to the prevention of metastatic gastric cancer, sponges containing 10 miR-598-3p binding sites were produced. This methodology presents numerous benefits for chemically modified, antisense, oligonucleotide inhibitors, particularly in investigations pertaining to long-term miRNA-interference [18, 19]. The findings of this experiment indicate that transfection with the miR-598-3p sponge resulted in a significant reduction in the expression of miR-598-3p (Fig. 3E). Additionally, the wound-healing assay revealed that miR-598-3p facilitated the migration of both SGC-7901 and MKN45 cells (Fig. 3A, B). Consequently, the transfection of the miR-598-3p sponge resulted in an increase in GC cell invasion, as evidenced by the findings in Fig. 3C and D. Furthermore, in vivo experiments demonstrated a significant elevation in the number of pulmonary metastatic nodes formed by GC cells expressing the miR-598-3p sponge compared to mice injected with GFP control cells (Fig. 3F, G). Additionally, the administration of GC cell-expressed sponges was associated with a shortened survival rate (Fig. 3J). Consistent with these observations, the sponge also promoted peritoneal metastasis (Fig. 3H, I). Overexpression of the sponge in GC cells resulted in a decrease in the expression of the epithelial marker E-cadherin under normal oxygen conditions. Additionally, the expression levels of the mesenchymal markers N-cadherin, Vimentin, and Fibronectin were found to be increased by the sponge (Fig. 3K). Furthermore, the loss of functional miR-598-3p was found to enhance the metastatic phenotype of gastric cancer, which aligns with previous findings.

### miR-598-3p directly targets RMP and IGF1r

**Fig. 4:**
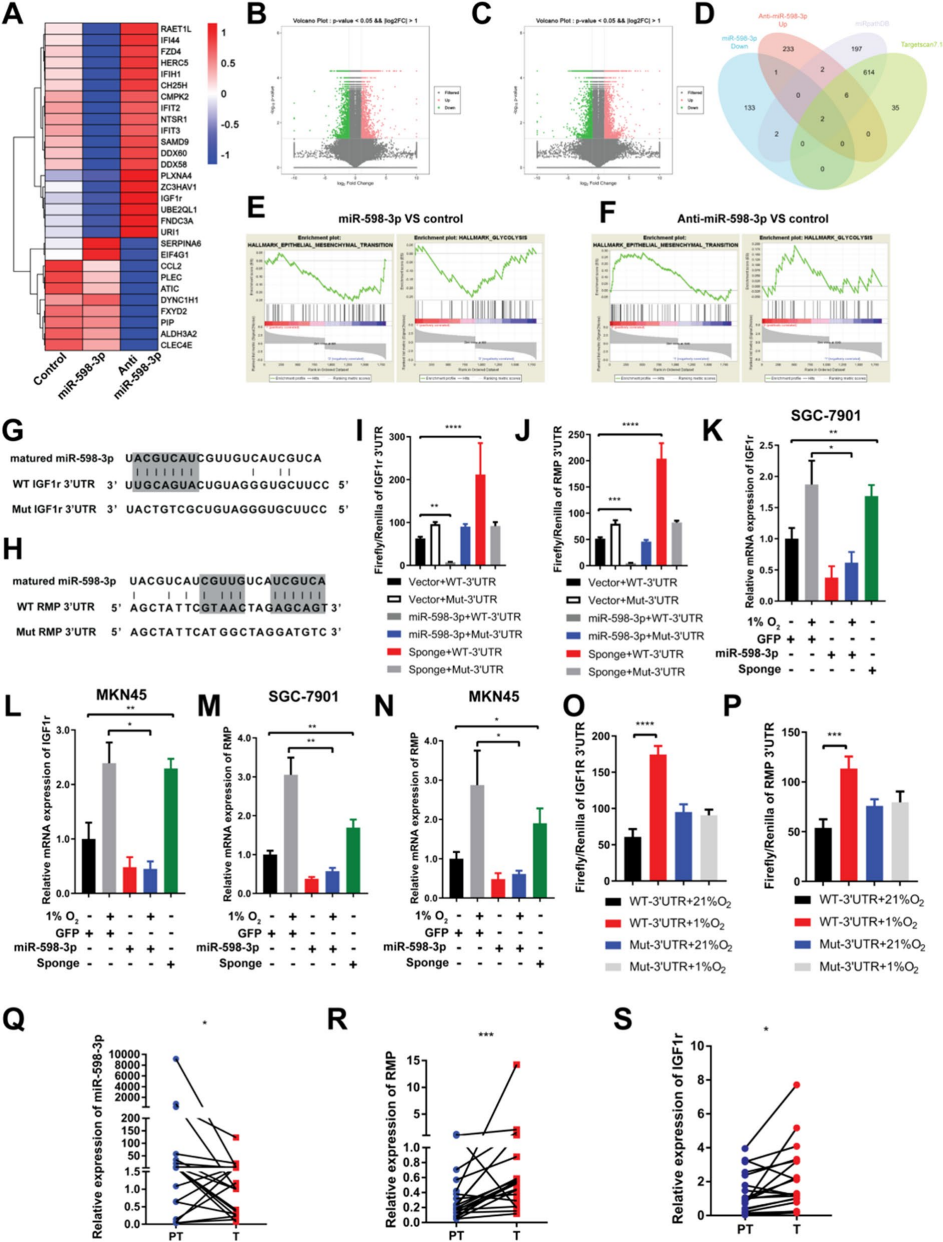
miR-598-3p directly targets RMP and IGF1r. **A** Heatmap showing differentially expressed genes in MKN45 cells with miR-598-3p overexpression (miR-598-3p) or knockdown (Anti miR-598-3p). **B**, **C** Volcano plot of miR-598-3p overexpression (**B**) or knockdown (**C**) versus control based on the RNA-seq analysis. **D** Venn diagram showing differentially expressed genes and prognostic genes derived from the indicated gene sets: miR-598-3p down (decreased gene sets in group of miR-598-3p in **A**), Anti-miR-598-3p up (increased gene sets in group of Anti-miR-598-3p in **A**), targeted genes predicted by miRpathDB or Targetscan 7.1. **E**, **F** Gene set enrichment analysis (GSEA) of the RNA-seq data set in **A**. **G**, **H** TargetScan 7.1 predicted the miR-598-3p binding sites in RMP and the IGF1r 3ʹUTR, as well as the designed mutant 3ʹUTR. **I**, **J** Luciferase reporter assay of the 3ʹUTR of RMP (**I**) or IGF1r (**J**) in HEK-293T cells cotransfected with the indicated plasmid. **K**–**N** mRNA expression of IGF1r and RMP in SGC-7901 or MKN45 cells that were cotransfected with the indicated plasmid, after which the cells were treated with or without hypoxia. **O**, **P** Luciferase reporter assay in HEK-293T cells cultured in hypoxia and transfected with wild-type (WT) or mutant (MUT) 3ʹUTR of RMP (**O**) and IGF1r (**P**). **Q**–**S** Real-time PCR analysis of miR-598-3p (**Q**), RMP (**R**) and IGF1r (**S**) levels in tumor tissue and relative paratumor tissue of 20 gastric cancer patients

To investigate the targets of miR-598-3p, we conducted transcriptome analysis on MKN45 cells under different conditions: control, miR-598-3p overexpression, and miR-598-3p knockdown. The resulting heatmap displays genes that exhibited at least a three fold change (Fig. 4A). Additionally, volcano diagrams were generated to visualize the differentially expressed genes, with a *p* value less than 0.05 and a log2FC greater than 1 (Fig. 4B, C). Subsequently, to further explore the potential targets involved in the hypoxia-driven metastasis of gastric cancer, we hypothesized that RMP and IGF1r could be targets of miR-598-3p based on the intersection of the 4 gene sets shown in the Venn diagrams (Fig. 4D). We further conducted gene set enrichment analysis (GSEA) and revealed that gene sets associated with EMT and glycolysis were observed in gastric cancer cells that either overexpressed or knocked down miR-598-3p (Fig. 4E, F). Additionally, we used TargetScan 7.1 to identify potential binding sites for miR-598-3p in the 3ʹ untranslated region (3'UTR) of IGF1r or RMP (Fig. 4G, H). To determine the direct targeting of IGF1r and RMP by miR-598-3p, a dual-luciferase reporter system was employed. In MKN45 cells, co-transfected the miR-598-3p mimics and dual-luciferase vector of wild-type 3ʹUTR of IGF1r or RMP resulted in a reduction in luciferase activity. Conversely, miR-598-3p sponge increased the luciferase activity of cell co-transfected with wild-type dual-luciferase vector (Fig. 4I, J). However, the luciferase activity in cells did not show a decrease or increase when the miR-598-3p mimic or sponge was co-transfected with the mutant IGF1r or RMP dual-luciferase vector (Fig. 4I, J). This observation suggests that miR-598-3p specifically and directly binds to the predicted binding site in the 3'UTR of IGF1r and RMP. Furthermore, HEK-293T cells were transfected with a dual-luciferase reporter of 3'UTR of IGF1r or RMP and subsequently cultured in either 21%O_2_ or 1%O_2_ for 12 h. The results indicate that the dual-luciferase reporter of wild-type IGF1r or RMP 3'UTR display higher activity than the mutant one under hypoxic conditions (Fig. 4O, P). Next, qRT‒PCR was conducted in SGC-7901 and MKN45 cells which transfected with miR-598-3p mimic or sponge. Followed by incubation in hypoxic conditions, the mRNA expression of IGF1r or RMP was notably increased, while the introduction of the miR-598-3p mimic effectively reversed this phenomenon (Fig. 4K–N). Furthermore, the expression of miR-598-3p, IGF1r, and RMP was conducted in a cohort comprising 20 cases of gastric cancer. These findings confirmed that both IGF1r and RMP exhibited high expression levels in gastric cancer tissues (T), whereas miR-598-3p was predominantly expressed in the paired adjacent normal tissues (PT) rather than cancer tissues (Fig. 4Q, R, S) which is consistent with our conjecture that hypoxia inhibits expression of miR-598-3p. Consequently, our results provide evidence that miR-598-3p directly targets both IGF1r and RMP.

### miR-598-3p suppressed glycolysis in GC cells by targeting RMP and IGF1r

**Fig. 5:**
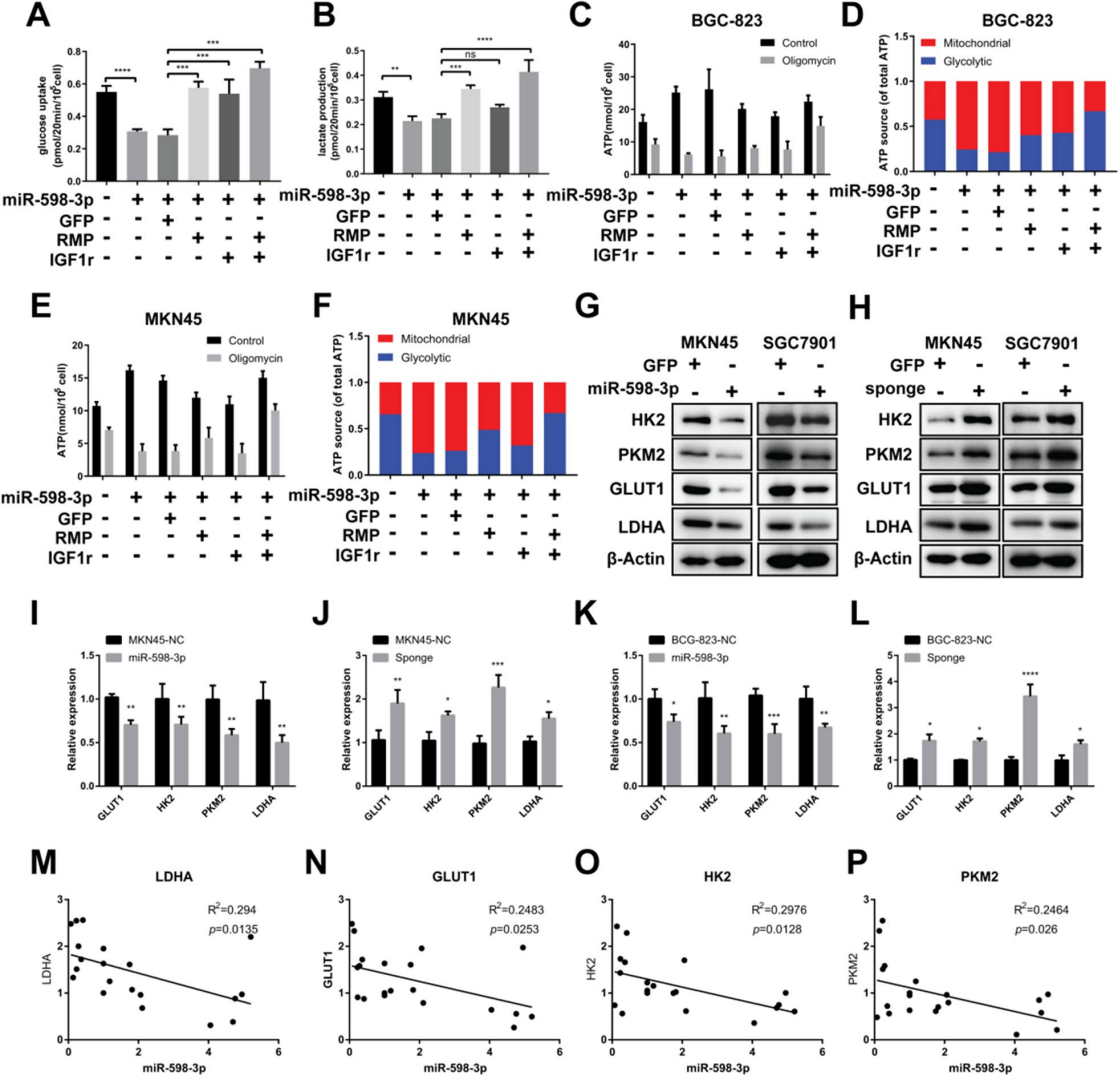
miR-598-3p suppressed glycolysis in GC cells by targeting RMP and IGF1r. **A**, **B** Glucose uptake (**A**) and lactate production (**B**) in MKN45 cells transfected with the indicated plasmid. **C**, **D** ATP production (**C**) and ATP source (**D**) of BGC-823 cells transfected with the indicated plasmid. **E**, **F** ATP production (**E**) and ATP source (**F**) of MKN45 cells transfected with the indicated plasmid. **G**, **H** Western blot analysis of glycolytic enzymes in MKN45 and BGC-823 cells overexpressing miR-598-3p (**G**) or the sponge (**H**). **I**–**L** Real-time qPCR analysis of GLUT1, HK2, PKM2 and LDHA expression in MKN45 and BGC-823 cells expressing miR-598-3p or the sponge. **M**–**P** Correlation analysis between miR-598-3p and LDHA (**M**), GLUT1 (**I**), HK2 (**J**) and PKM2 (**K**) expression in 20 GC samples

Next, we aimed to investigate the potential involvement of miR-598-3p and its targets IGF1r and RMP in glycolysis. To achieve this, mimic of miR-598-3p and vector of IGF1r, and RMP were co-transfected into MKN45 cells. The results showed that the introduction of the miR-598-3p mimic led to a significant reduction in glucose uptake and lactate production. Moreover, the overexpression of RMP and IGF1r, either individually or combination, was able to rescue the glucose consumption and lactate production that were repressed by miR-598-3p (Fig. 5A, B). Additionally, we examined the impact of miR-598-3p on ATP production and its source when glucose was provided as the sole carbon source in GC cells. The cellular ATP level was increased by overexpressing miR-598-3p in 2 GC cells and redundant by forced expression of IGF1r or RMP (Fig. 5C, E). Since overexpressing miR-598-3p in GC cell lines decreased glucose consumption and lactate production but increased ATP production, it is likely that there might exist a miR-598-3p-induced energy production switch from fermentation to respiration. To address this hypothesis, we treated cells with oligomycin, a mitochondrial ATPase inhibitor, and the results showed that 100 nM oligomycin treatment decreased ATP levels in GC cells. Significantly, the ATP level was observed to decrease to approximately one-fourth of its initial value following oligomycin treatment in cells overexpressing miR-598-3p (Fig. 5C, E). This finding suggests that a substantial proportion of ATP (approximately 80%) is generated through the respiratory pathway in cells with elevated miR-598-3p expression. Consistent with the above results, we found that ATP production was decreased when we forced IGF1r or RMP expression in miR-598-3p-overexpressing GC cells (Fig. 5C, E), subsequently leading to an increase in glycolytic ATP production (Fig. 5D, F). Furthermore, the miR-598-3p mimic exhibited a downregulatory effect on the mRNA and protein levels of glycolysis-related genes in GC cells (Fig. 5G, I, K). Conversely, overexpression of the miR-598-3p sponge resulted in an increase in the expression of glycolytic genes (Fig. 5H, J, L). To validate this finding, we conducted correlation analysis of the expression between miR-598-3p and glycolytic genes in a cohort of 20 GC patients. Consistently, miR-598-3p demonstrated a negative correlation with GLUT1, HK2, PKM2, and LDHA in these patients (Fig. 5M–P). Consequently, our results provide evidence that miR-598-3p disrupts hypoxic glycolysis by decreasing the expression of its targets RMP and IGF1r.

### miR-598-3p suppressed the metastasis of GC cells by inhibiting glycolysis

**Fig. 6:**
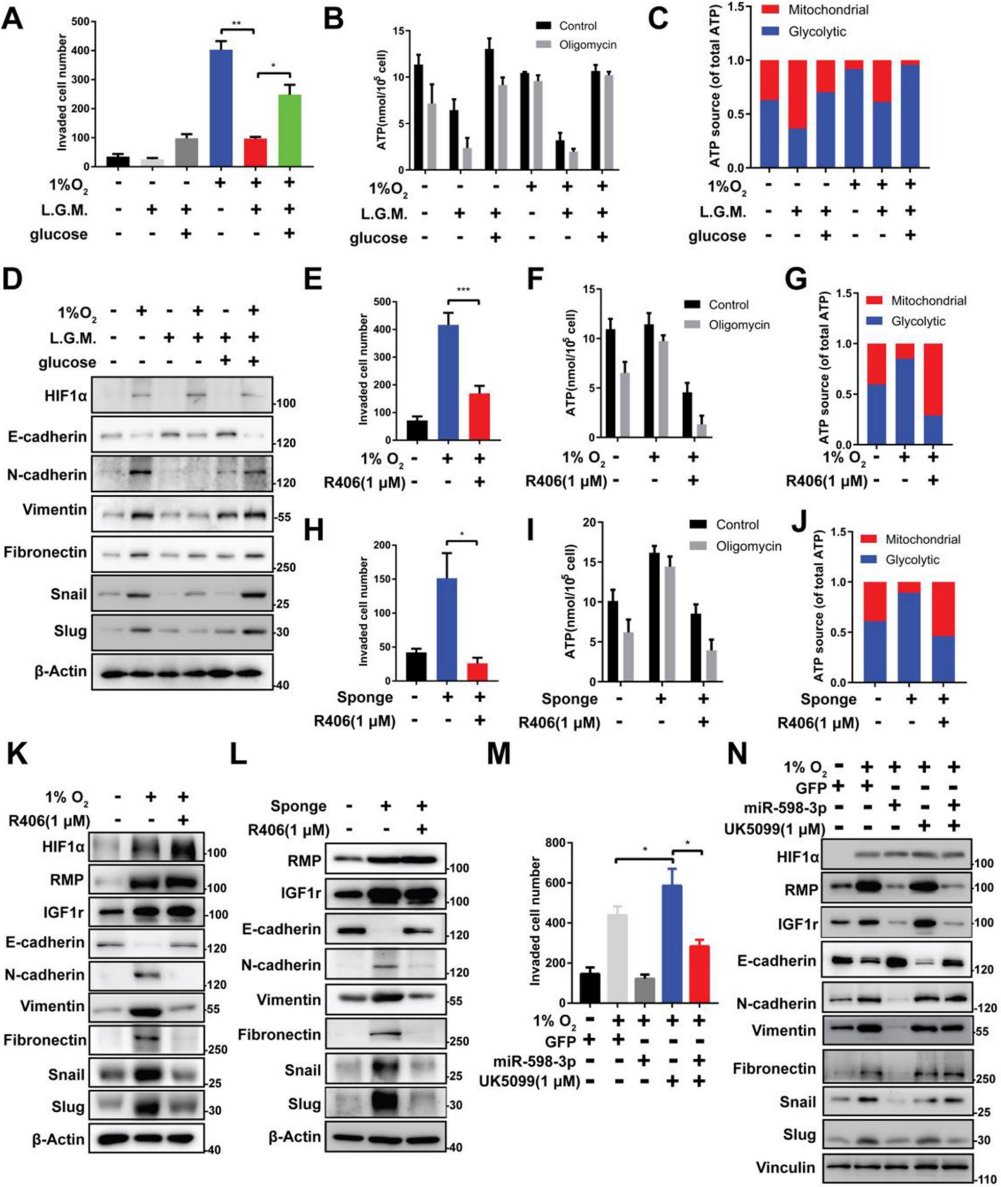
miR-598-3p suppressed the metastasis of GC cells by inhibiting glycolysis. **A** Transwell assay of hypoxic MKN45 cells cultured in low glucose medium (L.G.M.) 1.5 mg/L or L.G.M. supplemented with glucose to 4.5 mg/L. **B** ATP production of hypoxic MKN45 cells in **A**. **C** ATP source of hypoxic MKN45 cells in **A**. **D** Western blot analysis of MKN45 cells in **A**. **E** Transwell assay of MKN45 cells treated with hypoxia or 1 μM R406. **F** ATP production of hypoxic MKN45 cells in **F**. **G** ATP source of hypoxic MKN45 cells in **F**. **H** Transwell assay of MKN45 cells transfected with Sponge or treated with 1 μM R406. **I** ATP production of hypoxic MKN45 cells in **H**. **J** ATP source of hypoxic MKN45 cells in **H**. **K** Western blot analysis of cells in **E**. **L** Western blot analysis of cells in **H**. **M** Transwell assay of MKN45 cells transfected with miR-598-3p and then treated with hypoxia or 1 μM UK5099. **N** Western blot analysis of MKN45 cells in **M**

To explore whether the metabolic switch from oxidative phosphorylation to glycolysis promotes the metastasis of GC cells. We cultured MKN45 and BGC-823 cells with low glucose medium (L.G.M.). After 24 h, glucose uptake (Fig. S1A, B) and lactate production (Fig. S1G, H) were obstructed by L.G.M. and restored by supplementing glucose in GC cells. Under hypoxic conditions, glucose deprivation results in a decline in overall ATP production in GC cells. However, it is noteworthy that glucose deprivation can paradoxically enhance the rate of ATP production derived from mitochondria and promote cellular glucose utilization. Nevertheless, upon subsequent glucose supplementation, mitochondrial ATP synthesis significantly decrease, and prompting a shift towards glycolysis for energy generation (Fig. 6B, C). Consequently, a consistent provision of glucose is imperative to sustain glycolytic activity in hypoxic GC cells. Consistent with the decreased total cellular ATP and glycolytic ATP production, cell invasion and EMT markers were also reduced, whereas the reduced cell invasion and EMT markers were rescued by adding additional glucose (Fig. 6A, D). These findings suggest that hypoxia primarily stimulate the production of ATP through increased cellular glycolysis activity, thereby significantly hinder the expression of cellular EMT molecules, which is closely linked to cell metastasis (Fig. 6A). Next, we introduced R406, a compound that elicits a metabolic shift toward oxidative phosphorylation (OXPHOS) and induces an anti-Warburg effect. Hypoxic GC cells treated with 1 μM R406 exhibited decreased glucose uptake (Fig. S1C, D) and lactate production (Fig. S1I, J). More important, R406 obstructed glycolytic ATP production (Fig. 6F, G), which led to a decrease in EMT and metastasis during hypoxia (Fig. 6E, K). These data suggest that hypoxia mediated EMT and metastasis rely on the activity of glycolysis. To rule out the possibility that the metabolic shift was also involved in metastasis under normoxia. We treated cell with R406, the result shows miR-598-3p sponge-induced glucose uptake (Fig. S1E, F), cellular lactate production (Fig. S1K, L) and glycolytic ATP production (Fig. 6I, J) was significantly decreased in GC cells. Importantly, R406 efficiently reduced miR-598-3p depletion induced cell metastasis despite affecting the protein levels of RMP and IGF1r (Fig. 6H, L). Suggesting RMP and IGF1r trigger the metastatic phenotype by restraining miR-598-3p and liberating glycolysis. To further confirm these findings, we suppressed OXPHOS by UK5099, a potent mitochondrial pyruvate carrier inhibitor that promotes glycolysis by antagonizing pyruvate-driven mitochondrial respiration. With the remarkable enhancement of glycolysis, cell metastasis was abnormally increased by UK5099 in the presence of miR-598-3p during hypoxia (Fig. 6M, N). Taken together, these data clearly support that the metabolic transition toward glycolysis contributes to the metastatic phenotype under hypoxia, miR-598-3p reversed metastatic phenotype by increasing the percentage of ATP production from mitochondrial OXPHOS, eventually leads to a metabolic shift toward OXPHOS to avoid the malignant phenotypes of hypoxic GC cells.

### miR-598-3p elicits the anti-Warburg effect and its associated tumor metastasis by modulating RMP and IGF1r

**Fig. 7:**
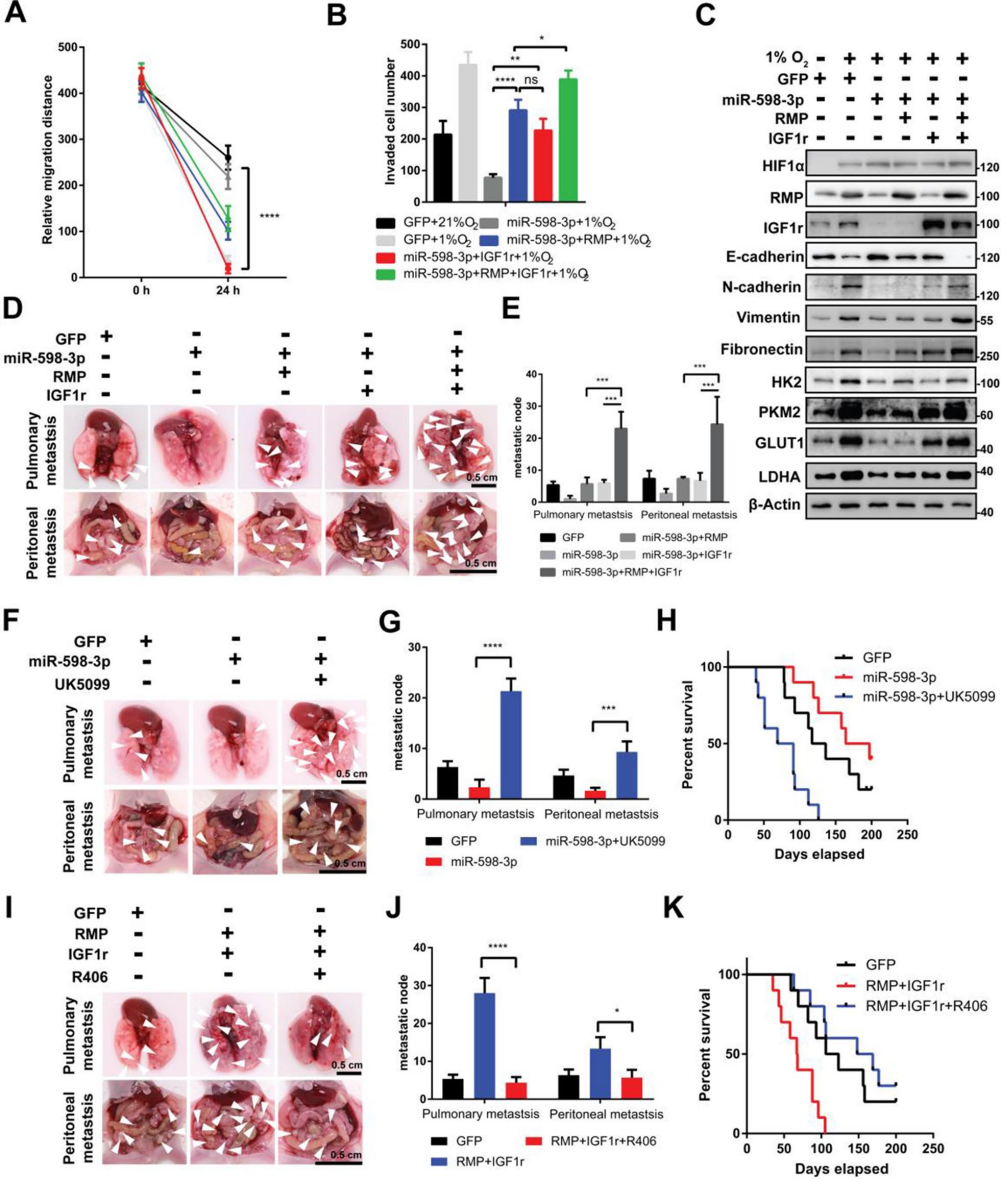
miR-598-3p elicits the anti-Warburg effect and associated tumor metastasis by modulating RMP and IGF1r in GC cells. **A**, **B** Wound-healing and transwell assays of hypoxia-cultured SGC-7901 cells transfected with the indicated plasmid. **C** Western blot analysis of hypoxic SGC-7901 cells transfected with the indicated plasmid. **D**, **E** BALB/c nude mice injected via the tail vein or intraperitoneally with SGC-7901 cells expressing the indicated plasmid. The white arrow depicts a metastatic node, n ≥ 5. **F**, **G** BALB/c nude mice injected via the tail vein or intraperitoneally with SGC-7901 cells transfected with GFP or miR-598-3p, after which cells were treated with or without UK5099. The white arrow depicts metastatic nodes, n ≥ 5. **H** Kaplan‒Meier survival curve of BALB/c nude mice injected via the tail vein with SGC-7901 cells transfected with GFP or miR-598-3p after 2 weeks of mice injected with or without UK5099 10 mg/kg/day. N = 10. **I**, **J** BALB/c nude mice injected via the tail vein or intraperitoneally with SGC-7901 cells expressing the indicated plasmid, after which cells were treated with or without R406. The white arrow depicts a metastatic node, n ≥ 5. **K** Kaplan‒Meier survival curve of BALB/c nude mice injected via the tail vein with SGC-7901 cells transfected with GFP, RMP or IGF1r after 2 weeks of injection with or without R406 (1 mg/kg/1 day). n ≥ 10

Subsequently, to validate the hypothesis that the reduction in metastasis was due to the downregulation of RMP and IGF1r, miR-598-3p was co-transfected with RMP and IGF1r into SGC-7901 cells under hypoxic conditions. The findings indicate that overexpression of either RMP or IGF1r, or both, effectively restored the diminished cell migration and invasion caused by miR-598-3p (Fig. 7A, B). Consistent with the metastatic behavior of the cells, a proportional increase in the protein levels of E-cadherin, N-cadherin, Vimentin, and Fibronectin was observed (Fig. 7C). Pulmonary and peritoneal metastasis assays showed that overexpression of RMP and IGF1r also showed a synergistic effect which significantly restored the number of metastatic nodes suppressed by miR-598-3p (Fig. 7D, E). These results indicate that miR-598-3p suppressed hypoxia-induced metastasis by specifically targeting RMP and IGF1r.

**Fig. 8:**
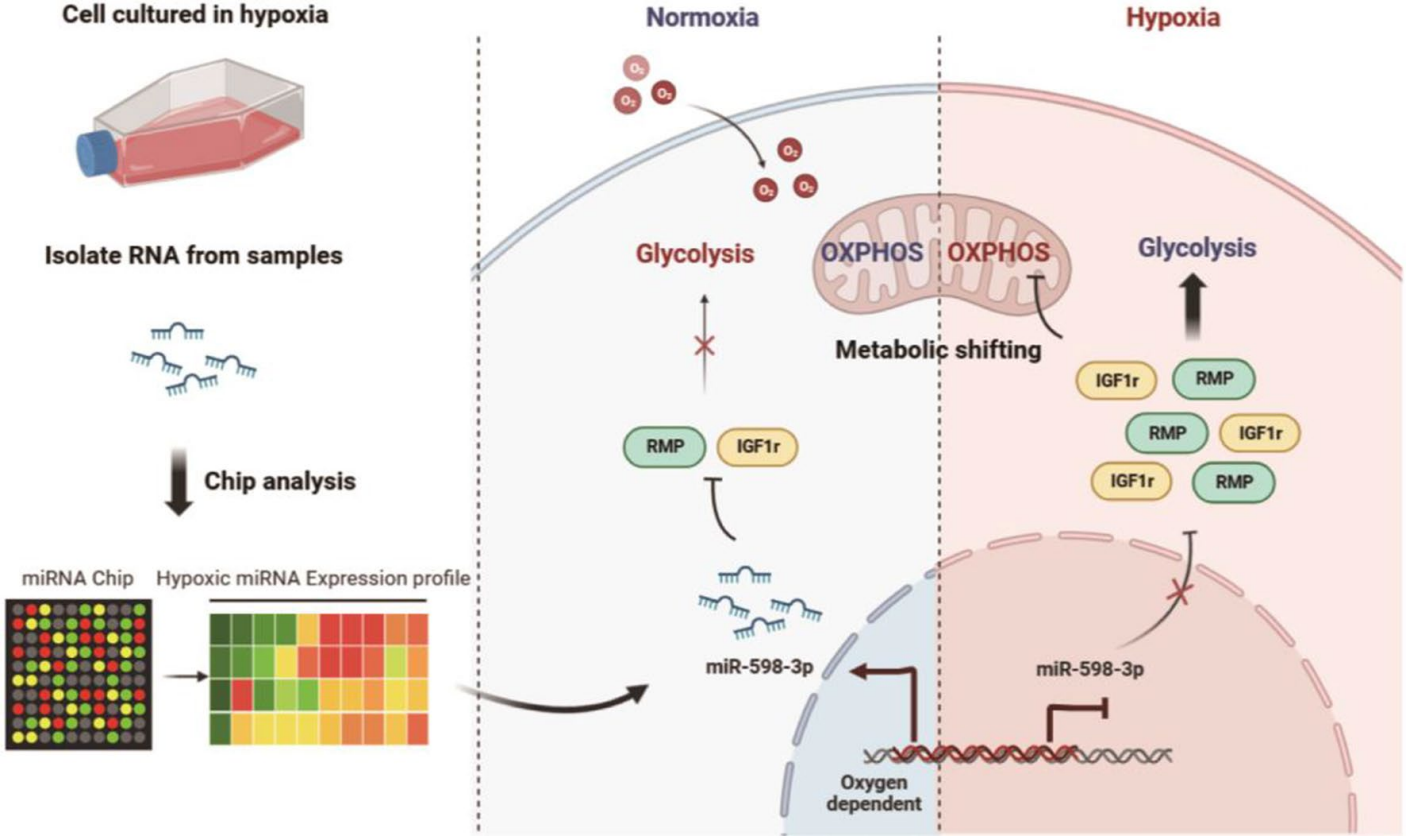
Model for hypoxia promotes metastasis by relieving miR-598-3p-restricted glycolysis in gastric cancer

The findings from our in vitro experiments provide strong evidence supporting beneficial effects of metabolic shifting on hypoxia-induced metastasis. To further investigate this possibility in vivo, we established a mouse model of pulmonary and peritoneal metastasis. Following tumor establishment, mice were treated with either vehicle or UK5099. As depicted in Fig. 7F and G, there was a significant reduction in the anti-metastatic effect of miR-598-3p upon administration of UK5099. Furthermore, a reduction in the survival rate was also observed in mice further treated with UK5099 (Fig. 7H). Next, a mouse model was administered with R406. Notably, R406 effectively mitigated the metastatic impact of RMP and IGF1r (Fig. 7I, J). Moreover, R406 significantly extended the survival of metastatic mice administered SGC-7901 cells overexpressing RMP and IGF1r (Fig. 7K). In summary, these findings highlight that the tumor suppressor miR-598-3p attenuates hypoxia-related metastasis and the metastatic colonization of distant organs by modulating the metabolic shift from glycolysis to OXPHOS in GC cells (Fig. 8).

## Discussion

Hypoxia is one of the main obstacles in treating solid tumors. Tumors growing under hypoxic conditions exhibit increased metastasis and enhanced resistance to conventional radiotherapy and chemotherapy [20]. In this study, we revealed the prognostic value and biological functions of miR-598-3p and its targets RMP and IGF1r in gastric cancer, which explained why and how hypoxic gastric cancer cells acquire a metastatic tendency toward glucose metabolic reprogramming. These alterations triggered metabolic remodeling and facilitated gastric cancer cells to leave oxygen-deficient sites to oxygen-rich sites to seek oxygen and energy, which also helped gastric cancer cell survival under metabolic stress.

The present research confirmed that miR-598 family was decreased in many solid tumors, such as CRC, lung cancer, osteosarcoma, and its downregulation was closely associated with the tumor cell proliferation, migration, invasion and self-renewal of cancer stem cells [13, 14, 21, 22]. The findings of our study indicate that hypoxia leads to the downregulation of miR-598-3p in GC cells. Furthermore, we elucidated that the repression of miR-598-3p in tumor microenvironment is not dependent on HIF1α. Previous studies have demonstrated that hypoxia induces EMT either through direct activation of notch signaling [23] or by promoting the expression of EMT factors mediated by HIF1α [24]. Theoretical frameworks for determining nutrient diffusion coefficients within tumors propose that the insufficiency of glucose and oxygen in the central regions of tumor nodules holds significant implications for the morphology and aggressiveness of tumors, aligning with the established impacts of hypoxia on tumors [25–27]. EMT is a vital process contributing to cancer progression, invasion, metastasis and characterized by the loss of epithelial markers and gain of mesenchymal markers. Most of mesenchymal markers were extracellular matrix component or in the interactions with adhesion receptors, the increased of mesenchymal markers have a remarkable impact on the biochemical and physical properties of the extracellular matrix dysregulation of cell adhesion and function, which is the first step of cancer metastasis. Clearly, the activation of EMT, especially through the upregulation of N-cadherin, Vimentin and Fibronectin by the suppression of miR-598-3p, have been shown to make a great contribution to gastric cancer metastasis in vitro and in vivo. Our research outcomes contribute novel insights into the mechanisms through which miR-598-3p hinders glycolysis, thereby inhibiting hypoxia-induced EMT and metastasis.

In investigating the inhibitory effects of miR-598-3p on glycolysis and associated EMT in GC cells, we identified RMP and IGF1r as crucial downstream targets of miR-598-3p. Our previous finding indicates that RMP interacts with RPB5 and possesses the ability to outcompete viral proteins or transcription factors that share the same binding site on RPB5 [28]. It was also reported that RMP promotes cancer cell proliferation in nutrient-rich conditions. However, RMP has been shown to decrease O-GlcNAcylation and enhance the turnover of c-MYC, enabling cells to adapt and survive in glucose-deprived environments [27], the underlining mechanism may expiating why RMP contribute to the adaptation of glucose-deprived environments of GC cell. Previous research has indicated that RMP exhibits oncogenic properties and is expressed in hepatocellular carcinoma (HCC). Additionally, the overexpression of RMP has been found to facilitate EMT, a known risk factor for metastasis [29]. The upregulation of *uri1* and *igf1r* genes is commonly observed in various cancer types, serving as a characteristic feature [30, 31]. Notably, IGF1r has been reported to play a crucial role in regulating cellular metabolism, proliferation, and survival, particularly in the context of cancer [32].

One of the most extensively studied modifications observed in neoplastic cells is a metabolic pathway adjustment in ATP generation, commonly referred to as the Warburg effect. This metabolic reprogramming is distinguished by the rapid production of ATP to meet the energy requirements of aberrantly proliferating cancer cells [33]. The production of ATP through aerobic glycolysis is estimated to occur approximately 10–100 times faster than mitochondrial oxidation [32]. However, it is intriguing to note that metastatic tumors exhibit higher energy consumption than primary tumors. Consequently, directing attention toward addressing the nutritional requirements of metastatic tumors may present a potentially effective approach in combating this perilous stage of cancer. Prior research has demonstrated that hypoxia triggers mitophagy, a process that eliminates impaired mitochondria and facilitates a metabolic shift toward glycolysis. This metabolic stress induces oxidative stress, thereby paradoxically promoting metastasis. The heightened OXPHOS activity leads to a substantial generation and buildup of reactive oxygen species (ROS), which ultimately triggers apoptosis [34]. However, the augmentation of glycolysis activity results in a substantial decrease in the generation of mitochondrial ROS, thereby facilitating cell survival and metastasis [35]. Previous finding suggests R406 reversed the Warburg effect by significantly deactivating Syk/PI3K pathway and UK5099 fueling glycolysis by specific inhibiting mitochondrial pyruvate carrier 1 (MPC-1) [34, 36, 37]. In our investigation, we unequivocally demonstrated that R406 induced the anti-Warburg effect in gastric cancer. The inhibition of glycolytic flux by R406 effectively impeded metastasis both in vitro and in vivo. Conversely, the administration of mitochondrial pyruvate carrier inhibitor UK5099, promotes glycolysis by counteracting the metabolic shift toward OXPHOS. Hypoxia-induced downregulation of miR-598-3p, along with the concerted actions of its downstream targets RMP and IGF1r, synergistically upregulated the expression of glycolytic enzymes HK2, GLUT1, and LDHA in hypoxic GC cells. This metabolic adaptation facilitated a metabolic switch from OXPHOS to glycolysis, enabling the maintenance of ATP production in the face of limited oxygen availability. Consequently, the reliance on glycolysis for ATP generation attenuated the generation of ROS from the mitochondrial electron transport chain. Current and future experimental and computational modeling of metastasis needs accommodated the plasticity of cancer metastasis, while integrating the complexity of microenvironment and many steps in the metastatic process. These stages encompass the initiation of metastasis and local invasion, travel to and colonization of distant metastatic sites, as well as the evasion of the immune system [38, 39]. Our study solely focuses on elucidating the initiation of metastasis under metabolic stress, disregarding the reciprocal influence of non-cancer cells present in hypoxic tumor microenvironment. Furthermore, the inquiry into the precise mechanisms through which miR-598-3p is downregulated under hypoxic conditions in GC cells necessitates further investigation.

In summary, our finding provides insights into the crucial roles of hypoxia in glucose metabolism and associated metastasis in gastric cancer. Specifically, miR-598-3p acts by targeting RMP and IGF1r to suppress the glycolytic pathway, thereby impeding the adaptive metabolic transition of GC cells. Regrettably, hypoxia within the tumor microenvironment consistently results in the downregulation of miR-598-3p. Through our investigation, we demonstrated that synthetic miR-598-3p can effectively inhibit glycolysis and associated metastasis which eventually enhanced overall survival. The results of our study offer a biological justification for employing a synthetic miR-598-3p mimic as an innovative therapeutic approach in addressing metastatic gastric carcinoma.

### Supplementary Information

**Additional file 1**: Sequence of primers and siRNA.

**Additional file 2**: Antibodies and componds.:

**Additional file 3**: Informed Consent.

**Additional file 4**: Additional method.

**Additional file 5**: **Figure S1. A and B**. Glucose uptake of hypoxic MKN45 (A) or BGC-823 (B) cells cultured in low glucose medium (L.G.M.) 1.5 mg/L or L.G.M. supplied with glucose to 4.5 mg/L. **C and D.** Glucose uptake of hypoxic MKN45 (C) or BGC-823 (D) cells treated with hypoxia or 1 μM R406. **E and F.** MKN45 (E) or BGC-823 (F) cells transfected with Sponge or treated with 1 μM R406. **G and H.** Lactate production of hypoxic MKN45 (G) or BGC-823 (H) cells cultured in low glucose medium (L.G.M.) 1.5 mg/L or L.G.M. supplied with glucose to 4.5 mg/L. **I and J.** Lactate production of hypoxic MKN45 (I) or BGC-823 (J) cells treated with hypoxia or 1 μM R406. **K and L.** Lactate production of MKN45 (K) or BGC-823 (L) cells transfected with Sponge or treated with 1 μM R406.

## Data Availability

The authors confirm that the data supporting the findings of this study are available within the article.
