# Supplementary material for: Hypoxia promotes metastasis by relieving miR-598-3p-restricted glycolysis in gastric cancer
Source: J Transl Med. 2024 Mar 15;22:283. doi: 10.1186/s12967-024-04957-7 (PMC10943772; doi:10.1186/s12967-024-04957-7)
Supplement: Supplementary file 3 — Additional file 3: Informed Consent. [file 12967_2024_4957_MOESM3_ESM.pdf]

临床研究受试者知情同意书

请您阅读以下材料，如果您愿意参加此项临床研究，您将了解此临床研究的性质以及如何参与其中。签署知情同意书将表明您已了解此临床研究并同意参加。按照我院医学伦理委员会要求在参与临床研究前签署知情同意书，这样可以保证您在了解临床研究的性质和参与的风险后再决定是否参与此临床研究。

1. 临床研究概述及目的

您被邀请参加我院的科研课题"胃癌患者肿瘤和癌旁组织中 miR-598-3p 表达"的临床调查。本研究是一项基础研究，目的是发掘促进胃癌发生发展的新指标。

2. 参加人员

（1）患者纳入标准：①术前未行任何抗肿瘤治疗及降期治疗术者；②行胃癌切除术；③术后病理诊断为胃癌患者。

（2）患者排除标准：①复发型或混合型胃癌；②术既往有恶性肿瘤病史者。

以上符合条件的患者将会参加这项研究。

3. 方法

胃癌患者肿瘤组织20例，配对的邻近非肿瘤组织20例。提取组织RNA，进行 miR-598-3p 和其他相关分子的荧光定量 PCR 检测。

4. 受益与风险

受试者可能从本项研究获得的受益：

（1）受试者将获得胃癌相关的危险因素教育；

（2）受试者可以获得与疾病相关的咨询服务。

本研究为横断面调查，仅分析病历数据，受试者承担的风险为最小风险。

5. 医疗与保护

本项临床研究的方案经过我院医学伦理委员会的审核，能很好的保护受试者的权益，并且具有科学性。参与本研究不会影响受试者的医疗方案和手段，本研究不涉及任何侵入性操作与药物试验，能很好的保护受试者的权益。

6. 自愿参加

您可以选择不参与此项临床研究，亦可以随时退出研究。

7. 保密

所有在调查中收集到的您的信息都将根据法律规定的程度进行保密。在研究记录中，您

将有一个标识编号。您的个人信息在没有您的书面许可的情况下是不会公布的。但是您的记录有可能被研究主办者，伦理委员会以及相关管理机构审查。此项试验的内容有可能发表，不过您的个人信息在任何刊物上都将是保密的。

8. 费用

参加此研究不会额外增加任何费用。

9. 研究者

研究者为检验科副研究员，合作者为肿瘤科具有丰富临床经验的副主任医师，在与您交流的过程的同时，也承担健康教育者的角色，最大程度保障受试者权益。

10. 权利

参加此项临床研究是自愿的。您可以选择不参加，或者您参加后可以随时退出。您的决定不会影响您目前或将来接受的治疗或其他服务。

有关调查研究的一般问题，请随时联系本项目的研究者。

11. 受试者声明

我已经仔细阅读上述关于临床研究的内容。我的提问均已得到满意回答。此表由我自愿签署，表明我参加此项研究的愿望。签署此同意书不能免除我的合法权利。如果有疑问，或受到与研究有关的伤害，我会与我的责任护士联系。

患者签名： 马秋芳　　　**日　　期：** 2022.2.26

联系电话： 13057169189

12. 临床研究者声明

我已经仔细的向受试者介绍了以上各项的情况。因此我确保我已用我个人所学的知识向受试者清楚地解释了临床研究的性质、要求、风险以及他/她签名的合法性。任何医学、语言或者教育的障碍都不会妨碍志愿者理解这些问题。

研究者签名： 周国　　　**联系电话：** 18862105653

日　　期： 2022.2.26

如果您有与自身权益相关的任何问题，或者您想反映参与本研究过程中的不满和忧虑，请联系伦理委员会办公室，联系电话：0519-68870261/68870201。

临床研究受试者知情同意书

请您阅读以下材料，如果您愿意参加此项临床研究，您将了解此临床研究的性质以及如何参与其中。签署知情同意书将表明您已了解此临床研究并同意参加。按照我院医学伦理委员会要求在参与临床研究前签署知情同意书，这样可以保证您在了解临床研究的性质和参与的风险后再决定是否参与此临床研究。

1. 临床研究概述及目的

您被邀请参加我院的科研课题“胃癌患者肿瘤和癌旁组织中 miR-598-3p 表达”的临床调查。本研究是一项基础研究，目的是发掘促进胃癌发生发展的新指标。

2. 参加人员

（1）患者纳入标准：①术前未行任何抗肿瘤治疗及降期治疗术者；②行胃癌切除术；③术后病理诊断为胃癌患者。

（2）患者排除标准：①复发型或混合型胃癌；②术既往有恶性肿瘤病史者。

以上符合条件的患者将会参加这项研究。

3. 方法

胃癌患者肿瘤组织20例，配对的邻近非肿瘤组织20例。提取组织RNA，进行 miR-598-3p 和其他相关分子的荧光定量 PCR 检测。

4. 受益与风险

受试者可能从本项研究获得的受益：

（1）受试者将获得胃癌相关的危险因素教育；

（2）受试者可以获得与疾病相关的咨询服务。

本研究为横断面调查，仅分析病历数据，受试者承担的风险为最小风险。

5. 医疗与保护

本项临床研究的方案经过我院医学伦理委员会的审核，能很好的保护受试者的权益，并且具有科学性。参与本研究不会影响受试者的医疗方案和手段，本研究不涉及任何侵入性操作与药物试验，能很好的保护受试者的权益。

6. 自愿参加

您可以选择不参与此项临床研究，亦可以随时退出研究。

7. 保密

所有在调查中收集到的您的信息都将根据法律规定的程度进行保密。在研究记录中，您

将有一个标识编号。您的个人信息在没有您的书面许可的情况下是不会公布的。但是您的记录有可能被研究主办者，伦理委员会以及相关管理机构审查。此项试验的内容有可能发表，不过您的个人信息在任何刊物上都将是保密的。

8. 费用

参加此研究不会额外增加任何费用。

9. 研究者

研究者为检验科副研究员，合作者为肿瘤科具有丰富临床经验的副主任医师，在与您交流的过程的同时，也承担健康教育者的角色，最大程度保障受试者权益。

10. 权利

参加此项临床研究是自愿的。您可以选择不参加，或者您参加后可以随时退出。您的决定不会影响您目前或将来接受的治疗或其他服务。

有关调查研究的一般问题，请随时联系本项目的研究者。

11. 受试者声明

我已经仔细阅读上述关于临床研究的内容。我的提问均已得到满意回答。此表由我自愿签署，表明我参加此项研究的愿望。签署此同意书不能免除我的合法权利。如果有疑问，或受到与研究有关的伤害，我会与我的责任护士联系。

患者签名：夏根祥　　　日　期：2022.02.26

联系电话：13585349662

12. 临床研究者声明

我已经仔细的向受试者介绍了以上各项的情况。因此我确保我已用我个人所学的知识向受试者清楚地解释了临床研究的性质、要求、风险以及他/她签名的合法性。任何医学、语言或者教育的障碍都不会妨碍志愿者理解这些问题。

研究者签名：[illegible]　　　联系电话：18862105653

日　期：2022.2.26

如果您有与自身权益相关的任何问题，或者您想反映参与本研究过程中的不满和忧虑，请联系伦理委员会办公室，联系电话：0519-68870261/68870201。

临床研究受试者知情同意书

请您阅读以下材料，如果您愿意参加此项临床研究，您将了解此临床研究的性质以及如何参与其中。签署知情同意书将表明您已了解此临床研究并同意参加。按照我院医学伦理委员会要求在参与临床研究前签署知情同意书，这样可以保证您在了解临床研究的性质和参与的风险后再决定是否参与此临床研究。

1. 临床研究概述及目的

您被邀请参加我院的科研课题"胃癌患者肿瘤和癌旁组织中 miR-598-3p 表达"的临床调查。本研究是一项基础研究，目的是发掘促进胃癌发生发展的新指标。

2. 参加人员

（1）患者纳入标准：①术前未行任何抗肿瘤治疗及降期治疗术者；②行胃癌切除术；③术后病理诊断为胃癌患者。

（2）患者排除标准：①复发型或混合型胃癌；②术既往有恶性肿瘤病史者。

以上符合条件的患者将会参加这项研究。

3. 方法

胃癌患者肿瘤组织 20 例，配对的邻近非肿瘤组织 20 例。提取组织 RNA，进行 miR-598-3p 和其他相关分子的荧光定量 PCR 检测。

4. 受益与风险

受试者可能从本项研究获得的受益：

（1）受试者将获得胃癌相关的危险因素教育；

（2）受试者可以获得与疾病相关的咨询服务。

本研究为横断面调查，仅分析病历数据，受试者承担的风险为最小风险。

5. 医疗与保护

本项临床研究的方案经过我院医学伦理委员会的审核，能很好的保护受试者的权益，并且具有科学性。参与本研究不会影响受试者的医疗方案和手段，本研究不涉及任何侵入性操作与药物试验，能很好的保护受试者的权益。

6. 自愿参加

您可以选择不参与此项临床研究，亦可以随时退出研究。

7. 保密

所有在调查中收集到的您的信息都将根据法律规定的程度进行保密。在研究记录中，您

将有一个标识编号。您的个人信息在没有您的书面许可的情况下是不会公布的。但是您的记录有可能被研究主办者，伦理委员会以及相关管理机构审查。此项试验的内容有可能发表，不过您的个人信息在任何刊物上都将是保密的。

8. 费用

参加此研究不会额外增加任何费用。

9. 研究者

研究者为检验科副研究员，合作者为肿瘤科具有丰富临床经验的副主任医师，在与您交流的过程的同时，也承担健康教育者的角色，最大程度保障受试者权益。

10. 权利

参加此项临床研究是自愿的。您可以选择不参加，或者您参加后可以随时退出。您的决定不会影响您目前或将来接受的治疗或其他服务。

有关调查研究的一般问题，请随时联系本项目的研究者。

11. 受试者声明

我已经仔细阅读上述关于临床研究的内容。我的提问均已得到满意回答。此表由我自愿签署，表明我参加此项研究的愿望。签署此同意书不能免除我的合法权利。如果有疑问，或受到与研究有关的伤害，我会与我的责任护士联系。

患者签名： 刘[illegible]清　　　　**日　　期：** 2022、2、26

联系电话： 86862991

12. 临床研究者声明

我已经仔细的向受试者介绍了以上各项的情况。因此我确保我已用我个人所学的知识向受试者清楚地解释了临床研究的性质、要求、风险以及他/她签名的合法性。任何医学、语言或者教育的障碍都不会妨碍志愿者理解这些问题。

研究者签名： 周[illegible]　　　　**联系电话：** 18862105653

日　　期： 2022.2.26

如果您有与自身权益相关的任何问题，或者您想反映参与本研究过程中的不满和忧虑，请联系伦理委员会办公室，联系电话：0519-68870261/68870201。

临床研究受试者知情同意书

请您阅读以下材料，如果您愿意参加此项临床研究，您将了解此临床研究的性质以及如何参与其中。签署知情同意书将表明您已了解此临床研究并同意参加。按照我院医学伦理委员会要求在参与临床研究前签署知情同意书，这样可以保证您在了解临床研究的性质和参与的风险后再决定是否参与此临床研究。

1. 临床研究概述及目的

您被邀请参加我院的科研课题“胃癌患者肿瘤和癌旁组织中 miR-598-3p 表达”的临床调查。本研究是一项基础研究，目的是发掘促进胃癌发生发展的新指标。

2. 参加人员

（1）患者纳入标准：①术前未行任何抗肿瘤治疗及降期治疗术者；②行胃癌切除术；③术后病理诊断为胃癌患者。

（2）患者排除标准：①复发型或混合型胃癌；②术既往有恶性肿瘤病史者。

以上符合条件的患者将会参加这项研究。

3. 方法

胃癌患者肿瘤组织20例，配对的邻近非肿瘤组织20例。提取组织RNA，进行 miR-598-3p 和其他相关分子的荧光定量 PCR 检测。

4. 受益与风险

受试者可能从本项研究获得的受益：

（1）受试者将获得胃癌相关的危险因素教育；

（2）受试者可以获得与疾病相关的咨询服务。

本研究为横断面调查，仅分析病历数据，受试者承担的风险为最小风险。

5. 医疗与保护

本项临床研究的方案经过我院医学伦理委员会的审核，能很好的保护受试者的权益，并且具有科学性。参与本研究不会影响受试者的医疗方案和手段，本研究不涉及任何侵入性操作与药物试验，能很好的保护受试者的权益。

6. 自愿参加

您可以选择不参与此项临床研究，亦可以随时退出研究。

7. 保密

所有在调查中收集到的您的信息都将根据法律规定的程度进行保密。在研究记录中，您

将有一个标识编号。您的个人信息在没有您的书面许可的情况下是不会公布的。但是您的记录有可能被研究主办者，伦理委员会以及相关管理机构审查。此项试验的内容有可能发表，不过您的个人信息在任何刊物上都将是保密的。

8. 费用

参加此研究不会额外增加任何费用。

9. 研究者

研究者为检验科副研究员，合作者为肿瘤科具有丰富临床经验的副主任医师，在与您交流的过程的同时，也承担健康教育者的角色，最大程度保障受试者权益。

10. 权利

参加此项临床研究是自愿的。您可以选择不参加，或者您参加后可以随时退出。您的决定不会影响您目前或将来接受的治疗或其他服务。

有关调查研究的一般问题，请随时联系本项目的研究者。

11. 受试者声明

我已经仔细阅读上述关于临床研究的内容。我的提问均已得到满意回答。此表由我自愿签署，表明我参加此项研究的愿望。签署此同意书不能免除我的合法权利。如果有疑问，或受到与研究有关的伤害，我会与我的责任护士联系。

患者签名： 陈江大　　　　**日　期：** 2022.2.26

联系电话： 13706115251

12. 临床研究者声明

我已经仔细的向受试者介绍了以上各项的情况。因此我确保我已用我个人所学的知识向受试者清楚地解释了临床研究的性质、要求、风险以及他/她签名的合法性。任何医学、语言或者教育的障碍都不会妨碍志愿者理解这些问题。

研究者签名： 周闻　　　　**联系电话：** 18862105653

日　　期： 2022.2.26

如果您有与自身权益相关的任何问题，或者您想反映参与本研究过程中的不满和忧虑，请联系伦理委员会办公室，联系电话：0519-68870261/68870201。

临床研究受试者知情同意书

请您阅读以下材料，如果您愿意参加此项临床研究，您将了解此临床研究的性质以及如何参与其中。签署知情同意书将表明您已了解此临床研究并同意参加。按照我院医学伦理委员会要求在参与临床研究前签署知情同意书，这样可以保证您在了解临床研究的性质和参与的风险后再决定是否参与此临床研究。

1. 临床研究概述及目的

您被邀请参加我院的科研课题“胃癌患者肿瘤和癌旁组织中 miR-598-3p 表达”的临床调查。本研究是一项基础研究，目的是发掘促进胃癌发生发展的新指标。

2. 参加人员

（1）患者纳入标准：①术前未行任何抗肿瘤治疗及降期治疗术者；②行胃癌切除术；③术后病理诊断为胃癌患者。

（2）患者排除标准：①复发型或混合型胃癌；②术既往有恶性肿瘤病史者。

以上符合条件的患者将会参加这项研究。

3. 方法

胃癌患者肿瘤组织 20 例，配对的邻近非肿瘤组织 20 例。提取组织 RNA，进行 miR-598-3p 和其他相关分子的荧光定量 PCR 检测。

4. 受益与风险

受试者可能从本项研究获得的受益：

（1）受试者将获得胃癌相关的危险因素教育；

（2）受试者可以获得与疾病相关的咨询服务。

本研究为横断面调查，仅分析病历数据，受试者承担的风险为最小风险。

5. 医疗与保护

本项临床研究的方案经过我院医学伦理委员会的审核，能很好的保护受试者的权益，并且具有科学性。参与本研究不会影响受试者的医疗方案和手段，本研究不涉及任何侵入性操作与药物试验，能很好的保护受试者的权益。

6. 自愿参加

您可以选择不参与此项临床研究，亦可以随时退出研究。

7. 保密

所有在调查中收集到的您的信息都将根据法律规定的程度进行保密。在研究记录中，您

将有一个标识编号。您的个人信息在没有您的书面许可的情况下是不会公布的。但是您的记录有可能被研究主办者，伦理委员会以及相关管理机构审查。此项试验的内容有可能发表，不过您的个人信息在任何刊物上都将是保密的。

8. 费用

参加此研究不会额外增加任何费用。

9. 研究者

研究者为检验科副研究员，合作者为肿瘤科具有丰富临床经验的副主任医师，在与您交流的过程的同时，也承担健康教育者的角色，最大程度保障受试者权益。

10. 权利

参加此项临床研究是自愿的。您可以选择不参加，或者您参加后可以随时退出。您的决定不会影响您目前或将来接受的治疗或其他服务。

有关调查研究的一般问题，请随时联系本项目的研究者。

11. 受试者声明

我已经仔细阅读上述关于临床研究的内容。我的提问均已得到满意回答。此表由我自愿签署，表明我参加此项研究的愿望。签署此同意书不能免除我的合法权利。如果有疑问，或受到与研究有关的伤害，我会与我的责任护士联系。

患者签名：[illegible]　　日　期：2022.2.21

联系电话：130[illegible]3156[illegible]21

12. 临床研究者声明

我已经仔细的向受试者介绍了以上各项的情况。因此我确保我已用我个人所学的知识向受试者清楚地解释了临床研究的性质、要求、风险以及他/她签名的合法性。任何医学、语言或者教育的障碍都不会妨碍志愿者理解这些问题。

研究者签名：[illegible]　　联系电话：18862105653

日　期：2022.2.21

如果您有与自身权益相关的任何问题，或者您想反映参与本研究过程中的不满和忧虑，请联系伦理委员会办公室，联系电话：0519-68870261/68870201。

临床研究受试者知情同意书

请您阅读以下材料，如果您愿意参加此项临床研究，您将了解此临床研究的性质以及如何参与其中。签署知情同意书将表明您已了解此临床研究并同意参加。按照我院医学伦理委员会要求在参与临床研究前签署知情同意书，这样可以保证您在了解临床研究的性质和参与的风险后再决定是否参与此临床研究。

1. 临床研究概述及目的

您被邀请参加我院的科研课题“胃癌患者肿瘤和癌旁组织中 miR-598-3p 表达”的临床调查。本研究是一项基础研究，目的是发掘促进胃癌发生发展的新指标。

2. 参加人员

（1）患者纳入标准：①术前未行任何抗肿瘤治疗及降期治疗术者；②行胃癌切除术；③术后病理诊断为胃癌患者。

（2）患者排除标准：①复发型或混合型胃癌；②术既往有恶性肿瘤病史者。

以上符合条件的患者将会参加这项研究。

3. 方法

胃癌患者肿瘤组织20例，配对的邻近非肿瘤组织20例。提取组织RNA，进行 miR-598-3p 和其他相关分子的荧光定量 PCR 检测。

4. 受益与风险

受试者可能从本项研究获得的受益：

（1）受试者将获得胃癌相关的危险因素教育；

（2）受试者可以获得与疾病相关的咨询服务。

本研究为横断面调查，仅分析病历数据，受试者承担的风险为最小风险。

5. 医疗与保护

本项临床研究的方案经过我院医学伦理委员会的审核，能很好的保护受试者的权益，并且具有科学性。参与本研究不会影响受试者的医疗方案和手段，本研究不涉及任何侵入性操作与药物试验，能很好的保护受试者的权益。

6. 自愿参加

您可以选择不参与此项临床研究，亦可以随时退出研究。

7. 保密

所有在调查中收集到的您的信息都将根据法律规定的程度进行保密。在研究记录中，您

将有一个标识编号。您的个人信息在没有您的书面许可的情况下是不会公布的。但是您的记录有可能被研究主办者，伦理委员会以及相关管理机构审查。此项试验的内容有可能发表，不过您的个人信息在任何刊物上都将是保密的。

8. 费用

参加此研究不会额外增加任何费用。

9. 研究者

研究者为检验科副研究员，合作者为肿瘤科具有丰富临床经验的副主任医师，在与您交流的过程的同时，也承担健康教育者的角色，最大程度保障受试者权益。

10. 权利

参加此项临床研究是自愿的。您可以选择不参加，或者您参加后可以随时退出。您的决定不会影响您目前或将来接受的治疗或其他服务。

有关调查研究的一般问题，请随时联系本项目的研究者。

11. 受试者声明

我已经仔细阅读上述关于临床研究的内容。我的提问均已得到满意回答。此表由我自愿签署，表明我参加此项研究的愿望。签署此同意书不能免除我的合法权利。如果有疑问，或受到与研究有关的伤害，我会与我的责任护士联系。

患者签名： 刘杏秀　　　　**日　　期：** 2022.12.15

联系电话： 13182503968

12. 临床研究者声明

我已经仔细的向受试者介绍了以上各项的情况。因此我确保我已用我个人所学的知识向受试者清楚地解释了临床研究的性质、要求、风险以及他/她签名的合法性。任何医学、语言或者教育的障碍都不会妨碍志愿者理解这些问题。

研究者签名： 周雨　　　　**联系电话：** 18862105653

日　　期： 2022.12.15

如果您有与自身权益相关的任何问题，或者您想反映参与本研究过程中的不满和忧虑，请联系伦理委员会办公室，联系电话：0519-68870261/68870201。

临床研究受试者知情同意书

请您阅读以下材料，如果您愿意参加此项临床研究，您将了解此临床研究的性质以及如何参与其中。签署知情同意书将表明您已了解此临床研究并同意参加。按照我院医学伦理委员会要求在参与临床研究前签署知情同意书，这样可以保证您在了解临床研究的性质和参与的风险后再决定是否参与此临床研究。

1. 临床研究概述及目的

您被邀请参加我院的科研课题“胃癌患者肿瘤和癌旁组织中 miR-598-3p 表达”的临床调查。本研究是一项基础研究，目的是发掘促进胃癌发生发展的新指标。

2. 参加人员

（1）患者纳入标准：①术前未行任何抗肿瘤治疗及降期治疗术者；②行胃癌切除术；③术后病理诊断为胃癌患者。

（2）患者排除标准：①复发型或混合型胃癌；②术既往有恶性肿瘤病史者。

以上符合条件的患者将会参加这项研究。

3. 方法

胃癌患者肿瘤组织20例，配对的邻近非肿瘤组织20例。提取组织RNA，进行 miR-598-3p 和其他相关分子的荧光定量 PCR 检测。

4. 受益与风险

受试者可能从本项研究获得的受益：

（1）受试者将获得胃癌相关的危险因素教育；

（2）受试者可以获得与疾病相关的咨询服务。

本研究为横断面调查，仅分析病历数据，受试者承担的风险为最小风险。

5. 医疗与保护

本项临床研究的方案经过我院医学伦理委员会的审核，能很好的保护受试者的权益，并且具有科学性。参与本研究不会影响受试者的医疗方案和手段，本研究不涉及任何侵入性操作与药物试验，能很好的保护受试者的权益。

6. 自愿参加

您可以选择不参与此项临床研究，亦可以随时退出研究。

7. 保密

所有在调查中收集到的您的信息都将根据法律规定的程度进行保密。在研究记录中，您

将有一个标识编号。您的个人信息在没有您的书面许可的情况下是不会公布的。但是您的记录有可能被研究主办者，伦理委员会以及相关管理机构审查。此项试验的内容有可能发表，不过您的个人信息在任何刊物上都将是保密的。

8. 费用

参加此研究不会额外增加任何费用。

9. 研究者

研究者为检验科副研究员，合作者为肿瘤科具有丰富临床经验的副主任医师，在与您交流的过程的同时，也承担健康教育者的角色，最大程度保障受试者权益。

10. 权利

参加此项临床研究是自愿的。您可以选择不参加，或者您参加后可以随时退出。您的决定不会影响您目前或将来接受的治疗或其他服务。

有关调查研究的一般问题，请随时联系本项目的研究者。

11. 受试者声明

我已经仔细阅读上述关于临床研究的内容。我的提问均已得到满意回答。此表由我自愿签署，表明我参加此项研究的愿望。签署此同意书不能免除我的合法权利。如果有疑问，或受到与研究有关的伤害，我会与我的责任护士联系。

患者签名：[illegible]　　日　期：2022.2.19.

联系电话：1340155 9115

12. 临床研究者声明

我已经仔细的向受试者介绍了以上各项的情况。因此我确保我已用我个人所学的知识向受试者清楚地解释了临床研究的性质、要求、风险以及他/她签名的合法性。任何医学、语言或者教育的障碍都不会妨碍志愿者理解这些问题。

研究者签名：[illegible]　　联系电话：18862105653

日　期：2022.2.19

如果您有与自身权益相关的任何问题，或者您想反映参与本研究过程中的不满和忧虑，请联系伦理委员会办公室，联系电话：0519-68870261/68870201。

临床研究受试者知情同意书

请您阅读以下材料，如果您愿意参加此项临床研究，您将了解此临床研究的性质以及如何参与其中。签署知情同意书将表明您已了解此临床研究并同意参加。按照我院医学伦理委员会要求在参与临床研究前签署知情同意书，这样可以保证您在了解临床研究的性质和参与的风险后再决定是否参与此临床研究。

1. 临床研究概述及目的

您被邀请参加我院的科研课题“胃癌患者肿瘤和癌旁组织中 miR-598-3p 表达”的临床调查。本研究是一项基础研究，目的是发掘促进胃癌发生发展的新指标。

2. 参加人员

（1）患者纳入标准：①术前未行任何抗肿瘤治疗及降期治疗术者；②行胃癌切除术；③术后病理诊断为胃癌患者。

（2）患者排除标准：①复发型或混合型胃癌；②术既往有恶性肿瘤病史者。

以上符合条件的患者将会参加这项研究。

3. 方法

胃癌患者肿瘤组织20例，配对的邻近非肿瘤组织20例。提取组织RNA，进行 miR-598-3p 和其他相关分子的荧光定量PCR检测。

4. 受益与风险

受试者可能从本项研究获得的受益：

（1）受试者将获得胃癌相关的危险因素教育；

（2）受试者可以获得与疾病相关的咨询服务。

本研究为横断面调查，仅分析病历数据，受试者承担的风险为最小风险。

5. 医疗与保护

本项临床研究的方案经过我院医学伦理委员会的审核，能很好的保护受试者的权益，并且具有科学性。参与本研究不会影响受试者的医疗方案和手段，本研究不涉及任何侵入性操作与药物试验，能很好的保护受试者的权益。

6. 自愿参加

您可以选择不参与此项临床研究，亦可以随时退出研究。

7. 保密

所有在调查中收集到的您的信息都将根据法律规定的程度进行保密。在研究记录中，您

将有一个标识编号。您的个人信息在没有您的书面许可的情况下是不会公布的。但是您的记录有可能被研究主办者，伦理委员会以及相关管理机构审查。此项试验的内容有可能发表，不过您的个人信息在任何刊物上都将是保密的。

8. 费用

参加此研究不会额外增加任何费用。

9. 研究者

研究者为检验科副研究员，合作者为肿瘤科具有丰富临床经验的副主任医师，在与您交流的过程的同时，也承担健康教育者的角色，最大程度保障受试者权益。

10. 权利

参加此项临床研究是自愿的。您可以选择不参加，或者您参加后可以随时退出。您的决定不会影响您目前或将来接受的治疗或其他服务。

有关调查研究的一般问题，请随时联系本项目的研究者。

11. 受试者声明

我已经仔细阅读上述关于临床研究的内容。我的提问均已得到满意回答。此表由我自愿签署，表明我参加此项研究的愿望。签署此同意书不能免除我的合法权利。如果有疑问，或受到与研究有关的伤害，我会与我的责任护士联系。

患者签名：刘相奇　　　　日　期：2022.2.19

联系电话：018962208128

12. 临床研究者声明

我已经仔细的向受试者介绍了以上各项的情况。因此我确保我已用我个人所学的知识向受试者清楚地解释了临床研究的性质、要求、风险以及他/她签名的合法性。任何医学、语言或者教育的障碍都不会妨碍志愿者理解这些问题。

研究者签名：[illegible]　　　　联系电话：18862105653

日　　期：2022.2.19

如果您有与自身权益相关的任何问题，或者您想反映参与本研究过程中的不满和忧虑，请联系伦理委员会办公室，联系电话：0519-68870261/68870201。

临床研究受试者知情同意书

请您阅读以下材料，如果您愿意参加此项临床研究，您将了解此临床研究的性质以及如何参与其中。签署知情同意书将表明您已了解此临床研究并同意参加。按照我院医学伦理委员会要求在参与临床研究前签署知情同意书，这样可以保证您在了解临床研究的性质和参与的风险后再决定是否参与此临床研究。

1. 临床研究概述及目的

您被邀请参加我院的科研课题“胃癌患者肿瘤和癌旁组织中 miR-598-3p 表达”的临床调查。本研究是一项基础研究，目的是发掘促进胃癌发生发展的新指标。

2. 参加人员

（1）患者纳入标准：①术前未行任何抗肿瘤治疗及降期治疗术者；②行胃癌切除术；③术后病理诊断为胃癌患者。

（2）患者排除标准：①复发型或混合型胃癌；②术既往有恶性肿瘤病史者。

以上符合条件的患者将会参加这项研究。

3. 方法

胃癌患者肿瘤组织20例，配对的邻近非肿瘤组织20例。提取组织RNA，进行 miR-598-3p 和其他相关分子的荧光定量 PCR 检测。

4. 受益与风险

受试者可能从本项研究获得的受益：

（1）受试者将获得胃癌相关的危险因素教育；

（2）受试者可以获得与疾病相关的咨询服务。

本研究为横断面调查，仅分析病历数据，受试者承担的风险为最小风险。

5. 医疗与保护

本项临床研究的方案经过我院医学伦理委员会的审核，能很好的保护受试者的权益，并且具有科学性。参与本研究不会影响受试者的医疗方案和手段，本研究不涉及任何侵入性操作与药物试验，能很好的保护受试者的权益。

6. 自愿参加

您可以选择不参与此项临床研究，亦可以随时退出研究。

7. 保密

所有在调查中收集到的您的信息都将根据法律规定的程度进行保密。在研究记录中，您

将有一个标识编号。您的个人信息在没有您的书面许可的情况下是不会公布的。但是您的记录有可能被研究主办者，伦理委员会以及相关管理机构审查。此项试验的内容有可能发表，不过您的个人信息在任何刊物上都将是保密的。

8. 费用

参加此研究不会额外增加任何费用。

9. 研究者

研究者为检验科副研究员，合作者为肿瘤科具有丰富临床经验的副主任医师，在与您交流的过程的同时，也承担健康教育者的角色，最大程度保障受试者权益。

10. 权利

参加此项临床研究是自愿的。您可以选择不参加，或者您参加后可以随时退出。您的决定不会影响您目前或将来接受的治疗或其他服务。

有关调查研究的一般问题，请随时联系本项目的研究者。

11. 受试者声明

我已经仔细阅读上述关于临床研究的内容。我的提问均已得到满意回答。此表由我自愿签署，表明我参加此项研究的愿望。签署此同意书不能免除我的合法权利。如果有疑问，或受到与研究有关的伤害，我会与我的责任护士联系。

患者签名：郑[illegible]大　　日　期：2022.2.16

联系电话：15351905615

12. 临床研究者声明

我已经仔细的向受试者介绍了以上各项的情况。因此我确保我已用我个人所学的知识向受试者清楚地解释了临床研究的性质、要求、风险以及他/她签名的合法性。任何医学、语言或者教育的障碍都不会妨碍志愿者理解这些问题。

研究者签名：周[illegible]　　联系电话：18862105653

日　期：2022.2.16

如果您有与自身权益相关的任何问题，或者您想反映参与本研究过程中的不满和忧虑，请联系伦理委员会办公室，联系电话：0519-68870261/68870201。

临床研究受试者知情同意书

请您阅读以下材料，如果您愿意参加此项临床研究，您将了解此临床研究的性质以及如何参与其中。签署知情同意书将表明您已了解此临床研究并同意参加。按照我院医学伦理委员会要求在参与临床研究前签署知情同意书，这样可以保证您在了解临床研究的性质和参与的风险后再决定是否参与此临床研究。

1. 临床研究概述及目的

您被邀请参加我院的科研课题“胃癌患者肿瘤和癌旁组织中 miR-598-3p 表达”的临床调查。本研究是一项基础研究，目的是发掘促进胃癌发生发展的新指标。

2. 参加人员

（1）患者纳入标准：①术前未行任何抗肿瘤治疗及降期治疗术者；②行胃癌切除术；③术后病理诊断为胃癌患者。

（2）患者排除标准：①复发型或混合型胃癌；②术既往有恶性肿瘤病史者。

以上符合条件的患者将会参加这项研究。

3. 方法

胃癌患者肿瘤组织20例，配对的邻近非肿瘤组织20例。提取组织RNA，进行 miR-598-3p 和其他相关分子的荧光定量 PCR 检测。

4. 受益与风险

受试者可能从本项研究获得的受益：

（1）受试者将获得胃癌相关的危险因素教育；

（2）受试者可以获得与疾病相关的咨询服务。

本研究为横断面调查，仅分析病历数据，受试者承担的风险为最小风险。

5. 医疗与保护

本项临床研究的方案经过我院医学伦理委员会的审核，能很好的保护受试者的权益，并且具有科学性。参与本研究不会影响受试者的医疗方案和手段，本研究不涉及任何侵入性操作与药物试验，能很好的保护受试者的权益。

6. 自愿参加

您可以选择不参与此项临床研究，亦可以随时退出研究。

7. 保密

所有在调查中收集到的您的信息都将根据法律规定的程度进行保密。在研究记录中，您

将有一个标识编号。您的个人信息在没有您的书面许可的情况下是不会公布的。但是您的记录有可能被研究主办者，伦理委员会以及相关管理机构审查。此项试验的内容有可能发表，不过您的个人信息在任何刊物上都将是保密的。

8. 费用

参加此研究不会额外增加任何费用。

9. 研究者

研究者为检验科副研究员，合作者为肿瘤科具有丰富临床经验的副主任医师，在与您交流的过程的同时，也承担健康教育者的角色，最大程度保障受试者权益。

10. 权利

参加此项临床研究是自愿的。您可以选择不参加，或者您参加后可以随时退出。您的决定不会影响您目前或将来接受的治疗或其他服务。

有关调查研究的一般问题，请随时联系本项目的研究者。

11. 受试者声明

我已经仔细阅读上述关于临床研究的内容。我的提问均已得到满意回答。此表由我自愿签署，表明我参加此项研究的愿望。签署此同意书不能免除我的合法权利。如果有疑问，或受到与研究有关的伤害，我会与我的责任护士联系。

患者签名： 张伯林　　　日　　期：2022.2.7

联系电话： 13063988878

12. 临床研究者声明

我已经仔细的向受试者介绍了以上各项的情况。因此我确保我已用我个人所学的知识向受试者清楚地解释了临床研究的性质、要求、风险以及他/她签名的合法性。任何医学、语言或者教育的障碍都不会妨碍志愿者理解这些问题。

研究者签名： [illegible]　　　**联系电话：** 18862105653

日　　期：2022.2.7

如果您有与自身权益相关的任何问题，或者您想反映参与本研究过程中的不满和忧虑，请联系伦理委员会办公室，联系电话：0519-68870261/68870201。

临床研究受试者知情同意书

请您阅读以下材料，如果您愿意参加此项临床研究，您将了解此临床研究的性质以及如何参与其中。签署知情同意书将表明您已了解此临床研究并同意参加。按照我院医学伦理委员会要求在参与临床研究前签署知情同意书，这样可以保证您在了解临床研究的性质和参与的风险后再决定是否参与此临床研究。

1. 临床研究概述及目的

您被邀请参加我院的科研课题“胃癌患者肿瘤和癌旁组织中 miR-598-3p 表达”的临床调查。本研究是一项基础研究，目的是发掘促进胃癌发生发展的新指标。

2. 参加人员

（1）患者纳入标准：①术前未行任何抗肿瘤治疗及降期治疗术者；②行胃癌切除术；③术后病理诊断为胃癌患者。

（2）患者排除标准：①复发型或混合型胃癌；②术既往有恶性肿瘤病史者。

以上符合条件的患者将会参加这项研究。

3. 方法

胃癌患者肿瘤组织20例，配对的邻近非肿瘤组织20例。提取组织RNA，进行 miR-598-3p 和其他相关分子的荧光定量 PCR 检测。

4. 受益与风险

受试者可能从本项研究获得的受益：

（1）受试者将获得胃癌相关的危险因素教育；

（2）受试者可以获得与疾病相关的咨询服务。

本研究为横断面调查，仅分析病历数据，受试者承担的风险为最小风险。

5. 医疗与保护

本项临床研究的方案经过我院医学伦理委员会的审核，能很好的保护受试者的权益，并且具有科学性。参与本研究不会影响受试者的医疗方案和手段，本研究不涉及任何侵入性操作与药物试验，能很好的保护受试者的权益。

6. 自愿参加

您可以选择不参与此项临床研究，亦可以随时退出研究。

7. 保密

所有在调查中收集到的您的信息都将根据法律规定的程度进行保密。在研究记录中，您

将有一个标识编号。您的个人信息在没有您的书面许可的情况下是不会公布的。但是您的记录有可能被研究主办者，伦理委员会以及相关管理机构审查。此项试验的内容有可能发表，不过您的个人信息在任何刊物上都将是保密的。

8. 费用

参加此研究不会额外增加任何费用。

9. 研究者

研究者为检验科副研究员，合作者为肿瘤科具有丰富临床经验的副主任医师，在与您交流的过程的同时，也承担健康教育者的角色，最大程度保障受试者权益。

10. 权利

参加此项临床研究是自愿的。您可以选择不参加，或者您参加后可以随时退出。您的决定不会影响您目前或将来接受的治疗或其他服务。

有关调查研究的一般问题，请随时联系本项目的研究者。

11. 受试者声明

我已经仔细阅读上述关于临床研究的内容。我的提问均已得到满意回答。此表由我自愿签署，表明我参加此项研究的愿望。签署此同意书不能免除我的合法权利。如果有疑问，或受到与研究有关的伤害，我会与我的责任护士联系。

患者签名： 洪凤梅　　　　**日　　期：** 2022.1.31

联系电话： 13961471388

12. 临床研究者声明

我已经仔细的向受试者介绍了以上各项的情况。因此我确保我已用我个人所学的知识向受试者清楚地解释了临床研究的性质、要求、风险以及他/她签名的合法性。任何医学、语言或者教育的障碍都不会妨碍志愿者理解这些问题。

研究者签名： 周雨　　　　**联系电话：** 18862105653

日　　期： 2022.1.31

如果您有与自身权益相关的任何问题，或者您想反映参与本研究过程中的不满和忧虑，请联系伦理委员会办公室，联系电话：0519-68870261/68870201。

临床研究受试者知情同意书

请您阅读以下材料，如果您愿意参加此项临床研究，您将了解此临床研究的性质以及如何参与其中。签署知情同意书将表明您已了解此临床研究并同意参加。按照我院医学伦理委员会要求在参与临床研究前签署知情同意书，这样可以保证您在了解临床研究的性质和参与的风险后再决定是否参与此临床研究。

1. 临床研究概述及目的

您被邀请参加我院的科研课题“胃癌患者肿瘤和癌旁组织中 miR-598-3p 表达”的临床调查。本研究是一项基础研究，目的是发掘促进胃癌发生发展的新指标。

2. 参加人员

（1）患者纳入标准：①术前未行任何抗肿瘤治疗及降期治疗术者；②行胃癌切除术；③术后病理诊断为胃癌患者。

（2）患者排除标准：①复发型或混合型胃癌；②术既往有恶性肿瘤病史者。

以上符合条件的患者将会参加这项研究。

3. 方法

胃癌患者肿瘤组织20例，配对的邻近非肿瘤组织20例。提取组织RNA，进行 miR-598-3p 和其他相关分子的荧光定量 PCR 检测。

4. 受益与风险

受试者可能从本项研究获得的受益：

（1）受试者将获得胃癌相关的危险因素教育；

（2）受试者可以获得与疾病相关的咨询服务。

本研究为横断面调查，仅分析病历数据，受试者承担的风险为最小风险。

5. 医疗与保护

本项临床研究的方案经过我院医学伦理委员会的审核，能很好的保护受试者的权益，并且具有科学性。参与本研究不会影响受试者的医疗方案和手段，本研究不涉及任何侵入性操作与药物试验，能很好的保护受试者的权益。

6. 自愿参加

您可以选择不参与此项临床研究，亦可以随时退出研究。

7. 保密

所有在调查中收集到的您的信息都将根据法律规定的程度进行保密。在研究记录中，您

将有一个标识编号。您的个人信息在没有您的书面许可的情况下是不会公布的。但是您的记录有可能被研究主办者，伦理委员会以及相关管理机构审查。此项试验的内容有可能发表，不过您的个人信息在任何刊物上都将是保密的。

8. 费用

参加此研究不会额外增加任何费用。

9. 研究者

研究者为检验科副研究员，合作者为肿瘤科具有丰富临床经验的副主任医师，在与您交流的过程的同时，也承担健康教育者的角色，最大程度保障受试者权益。

10. 权利

参加此项临床研究是自愿的。您可以选择不参加，或者您参加后可以随时退出。您的决定不会影响您目前或将来接受的治疗或其他服务。

有关调查研究的一般问题，请随时联系本项目的研究者。

11. 受试者声明

我已经仔细阅读上述关于临床研究的内容。我的提问均已得到满意回答。此表由我自愿签署，表明我参加此项研究的愿望。签署此同意书不能免除我的合法权利。如果有疑问，或受到与研究有关的伤害，我会与我的责任护士联系。

患者签名： 余金祥　　**日　期：** 2022.1.29

联系电话： 13701500185

12. 临床研究者声明

我已经仔细的向受试者介绍了以上各项的情况。因此我确保我已用我个人所学的知识向受试者清楚地解释了临床研究的性质、要求、风险以及他/她签名的合法性。任何医学、语言或者教育的障碍都不会妨碍志愿者理解这些问题。

研究者签名： 周雨　　**联系电话：** 18862105653

日　期： 2022.1.29

如果您有与自身权益相关的任何问题，或者您想反映参与本研究过程中的不满和忧虑，请联系伦理委员会办公室，联系电话：0519-68870261/68870201。

临床研究受试者知情同意书

请您阅读以下材料，如果您愿意参加此项临床研究，您将了解此临床研究的性质以及如何参与其中。签署知情同意书将表明您已了解此临床研究并同意参加。按照我院医学伦理委员会要求在参与临床研究前签署知情同意书，这样可以保证您在了解临床研究的性质和参与的风险后再决定是否参与此临床研究。

1. 临床研究概述及目的

您被邀请参加我院的科研课题“胃癌患者肿瘤和癌旁组织中 miR-598-3p 表达”的临床调查。本研究是一项基础研究，目的是发掘促进胃癌发生发展的新指标。

2. 参加人员

（1）患者纳入标准：①术前未行任何抗肿瘤治疗及降期治疗术者；②行胃癌切除术；③术后病理诊断为胃癌患者。

（2）患者排除标准：①复发型或混合型胃癌；②术既往有恶性肿瘤病史者。

以上符合条件的患者将会参加这项研究。

3. 方法

胃癌患者肿瘤组织20例，配对的邻近非肿瘤组织20例。提取组织RNA，进行 miR-598-3p 和其他相关分子的荧光定量 PCR 检测。

4. 受益与风险

受试者可能从本项研究获得的受益：

（1）受试者将获得胃癌相关的危险因素教育；

（2）受试者可以获得与疾病相关的咨询服务。

本研究为横断面调查，仅分析病历数据，受试者承担的风险为最小风险。

5. 医疗与保护

本项临床研究的方案经过我院医学伦理委员会的审核，能很好的保护受试者的权益，并且具有科学性。参与本研究不会影响受试者的医疗方案和手段，本研究不涉及任何侵入性操作与药物试验，能很好的保护受试者的权益。

6. 自愿参加

您可以选择不参与此项临床研究，亦可以随时退出研究。

7. 保密

所有在调查中收集到的您的信息都将根据法律规定的程度进行保密。在研究记录中，您

将有一个标识编号。您的个人信息在没有您的书面许可的情况下是不会公布的。但是您的记录有可能被研究主办者，伦理委员会以及相关管理机构审查。此项试验的内容有可能发表，不过您的个人信息在任何刊物上都将是保密的。

8. 费用

参加此研究不会额外增加任何费用。

9. 研究者

研究者为检验科副研究员，合作者为肿瘤科具有丰富临床经验的副主任医师，在与您交流的过程的同时，也承担健康教育者的角色，最大程度保障受试者权益。

10. 权利

参加此项临床研究是自愿的。您可以选择不参加，或者您参加后可以随时退出。您的决定不会影响您目前或将来接受的治疗或其他服务。

有关调查研究的一般问题，请随时联系本项目的研究者。

11. 受试者声明

我已经仔细阅读上述关于临床研究的内容。我的提问均已得到满意回答。此表由我自愿签署，表明我参加此项研究的愿望。签署此同意书不能免除我的合法权利。如果有疑问，或受到与研究有关的伤害，我会与我的责任护士联系。

患者签名： 沈国荣　　　　**日　　期：** 2022. 1. 29

联系电话： 0511 - 86454122

12. 临床研究者声明

我已经仔细的向受试者介绍了以上各项的情况。因此我确保我已用我个人所学的知识向受试者清楚地解释了临床研究的性质、要求、风险以及他/她签名的合法性。任何医学、语言或者教育的障碍都不会妨碍志愿者理解这些问题。

研究者签名： [illegible]　　　　**联系电话：** 18862105653

日　　期： 2022. 1. 29

如果您有与自身权益相关的任何问题，或者您想反映参与本研究过程中的不满和忧虑，请联系伦理委员会办公室，联系电话：0519-68870261/68870201。

临床研究受试者知情同意书

请您阅读以下材料，如果您愿意参加此项临床研究，您将了解此临床研究的性质以及如何参与其中。签署知情同意书将表明您已了解此临床研究并同意参加。按照我院医学伦理委员会要求在参与临床研究前签署知情同意书，这样可以保证您在了解临床研究的性质和参与的风险后再决定是否参与此临床研究。

1. 临床研究概述及目的

您被邀请参加我院的科研课题“胃癌患者肿瘤和癌旁组织中 miR-598-3p 表达”的临床调查。本研究是一项基础研究，目的是发掘促进胃癌发生发展的新指标。

2. 参加人员

（1）患者纳入标准：①术前未行任何抗肿瘤治疗及降期治疗术者；②行胃癌切除术；③术后病理诊断为胃癌患者。

（2）患者排除标准：①复发型或混合型胃癌；②术既往有恶性肿瘤病史者。

以上符合条件的患者将会参加这项研究。

3. 方法

胃癌患者肿瘤组织20例，配对的邻近非肿瘤组织20例。提取组织RNA，进行 miR-598-3p 和其他相关分子的荧光定量 PCR 检测。

4. 受益与风险

受试者可能从本项研究获得的受益：

（1）受试者将获得胃癌相关的危险因素教育；

（2）受试者可以获得与疾病相关的咨询服务。

本研究为横断面调查，仅分析病历数据，受试者承担的风险为最小风险。

5. 医疗与保护

本项临床研究的方案经过我院医学伦理委员会的审核，能很好的保护受试者的权益，并且具有科学性。参与本研究不会影响受试者的医疗方案和手段，本研究不涉及任何侵入性操作与药物试验，能很好的保护受试者的权益。

6. 自愿参加

您可以选择不参与此项临床研究，亦可以随时退出研究。

7. 保密

所有在调查中收集到的您的信息都将根据法律规定的程度进行保密。在研究记录中，您

将有一个标识编号。您的个人信息在没有您的书面许可的情况下是不会公布的。但是您的记录有可能被研究主办者，伦理委员会以及相关管理机构审查。此项试验的内容有可能发表，不过您的个人信息在任何刊物上都将是保密的。

8. 费用

参加此研究不会额外增加任何费用。

9. 研究者

研究者为检验科副研究员，合作者为肿瘤科具有丰富临床经验的副主任医师，在与您交流的过程的同时，也承担健康教育者的角色，最大程度保障受试者权益。

10. 权利

参加此项临床研究是自愿的。您可以选择不参加，或者您参加后可以随时退出。您的决定不会影响您目前或将来接受的治疗或其他服务。

有关调查研究的一般问题，请随时联系本项目的研究者。

11. 受试者声明

我已经仔细阅读上述关于临床研究的内容。我的提问均已得到满意回答。此表由我自愿签署，表明我参加此项研究的愿望。签署此同意书不能免除我的合法权利。如果有疑问，或受到与研究有关的伤害，我会与我的责任护士联系。

患者签名：[illegible]　　日　　期：2022.1.29

联系电话：13391926269

12. 临床研究者声明

我已经仔细的向受试者介绍了以上各项的情况。因此我确保我已用我个人所学的知识向受试者清楚地解释了临床研究的性质、要求、风险以及他/她签名的合法性。任何医学、语言或者教育的障碍都不会妨碍志愿者理解这些问题。

研究者签名：[illegible]　　联系电话：18862105653

日　　期：2022.1.29

如果您有与自身权益相关的任何问题，或者您想反映参与本研究过程中的不满和忧虑，请联系伦理委员会办公室，联系电话：0519-68870261/68870201。

临床研究受试者知情同意书

请您阅读以下材料，如果您愿意参加此项临床研究，您将了解此临床研究的性质以及如何参与其中。签署知情同意书将表明您已了解此临床研究并同意参加。按照我院医学伦理委员会要求在参与临床研究前签署知情同意书，这样可以保证您在了解临床研究的性质和参与的风险后再决定是否参与此临床研究。

1. 临床研究概述及目的

您被邀请参加我院的科研课题“胃癌患者肿瘤和癌旁组织中 miR-598-3p 表达”的临床调查。本研究是一项基础研究，目的是发掘促进胃癌发生发展的新指标。

2. 参加人员

（1）患者纳入标准：①术前未行任何抗肿瘤治疗及降期治疗术者；②行胃癌切除术；③术后病理诊断为胃癌患者。

（2）患者排除标准：①复发型或混合型胃癌；②术既往有恶性肿瘤病史者。

以上符合条件的患者将会参加这项研究。

3. 方法

胃癌患者肿瘤组织 20 例，配对的邻近非肿瘤组织 20 例。提取组织 RNA，进行 miR-598-3p 和其他相关分子的荧光定量 PCR 检测。

4. 受益与风险

受试者可能从本项研究获得的受益：

（1）受试者将获得胃癌相关的危险因素教育；

（2）受试者可以获得与疾病相关的咨询服务。

本研究为横断面调查，仅分析病历数据，受试者承担的风险为最小风险。

5. 医疗与保护

本项临床研究的方案经过我院医学伦理委员会的审核，能很好的保护受试者的权益，并且具有科学性。参与本研究不会影响受试者的医疗方案和手段，本研究不涉及任何侵入性操作与药物试验，能很好的保护受试者的权益。

6. 自愿参加

您可以选择不参与此项临床研究，亦可以随时退出研究。

7. 保密

所有在调查中收集到的您的信息都将根据法律规定的程度进行保密。在研究记录中，您

将有一个标识编号。您的个人信息在没有您的书面许可的情况下是不会公布的。但是您的记录有可能被研究主办者，伦理委员会以及相关管理机构审查。此项试验的内容有可能发表，不过您的个人信息在任何刊物上都将是保密的。

8. 费用

参加此研究不会额外增加任何费用。

9. 研究者

研究者为检验科副研究员，合作者为肿瘤科具有丰富临床经验的副主任医师，在与您交流的过程的同时，也承担健康教育者的角色，最大程度保障受试者权益。

10. 权利

参加此项临床研究是自愿的。您可以选择不参加，或者您参加后可以随时退出。您的决定不会影响您目前或将来接受的治疗或其他服务。

有关调查研究的一般问题，请随时联系本项目的研究者。

11. 受试者声明

我已经仔细阅读上述关于临床研究的内容。我的提问均已得到满意回答。此表由我自愿签署，表明我参加此项研究的愿望。签署此同意书不能免除我的合法权利。如果有疑问，或受到与研究有关的伤害，我会与我的责任护士联系。

患者签名：魏春桃　　日　　期：2022.1.19

联系电话：15150545708

12. 临床研究者声明

我已经仔细的向受试者介绍了以上各项的情况。因此我确保我已用我个人所学的知识向受试者清楚地解释了临床研究的性质、要求、风险以及他/她签名的合法性。任何医学、语言或者教育的障碍都不会妨碍志愿者理解这些问题。

研究者签名：[illegible]　　联系电话：18862105653

日　　期：2022.1.19

如果您有与自身权益相关的任何问题，或者您想反映参与本研究过程中的不满和忧虑，请联系伦理委员会办公室，联系电话：0519-68870261/68870201。

临床研究受试者知情同意书

请您阅读以下材料，如果您愿意参加此项临床研究，您将了解此临床研究的性质以及如何参与其中。签署知情同意书将表明您已了解此临床研究并同意参加。按照我院医学伦理委员会要求在参与临床研究前签署知情同意书，这样可以保证您在了解临床研究的性质和参与的风险后再决定是否参与此临床研究。

1. 临床研究概述及目的

您被邀请参加我院的科研课题“胃癌患者肿瘤和癌旁组织中 miR-598-3p 表达”的临床调查。本研究是一项基础研究，目的是发掘促进胃癌发生发展的新指标。

2. 参加人员

（1）患者纳入标准：①术前未行任何抗肿瘤治疗及降期治疗术者；②行胃癌切除术；③术后病理诊断为胃癌患者。

（2）患者排除标准：①复发型或混合型胃癌；②术既往有恶性肿瘤病史者。

以上符合条件的患者将会参加这项研究。

3. 方法

胃癌患者肿瘤组织20例，配对的邻近非肿瘤组织20例。提取组织RNA，进行 miR-598-3p 和其他相关分子的荧光定量 PCR 检测。

4. 受益与风险

受试者可能从本项研究获得的受益：

（1）受试者将获得胃癌相关的危险因素教育；

（2）受试者可以获得与疾病相关的咨询服务。

本研究为横断面调查，仅分析病历数据，受试者承担的风险为最小风险。

5. 医疗与保护

本项临床研究的方案经过我院医学伦理委员会的审核，能很好的保护受试者的权益，并且具有科学性。参与本研究不会影响受试者的医疗方案和手段，本研究不涉及任何侵入性操作与药物试验，能很好的保护受试者的权益。

6. 自愿参加

您可以选择不参与此项临床研究，亦可以随时退出研究。

7. 保密

所有在调查中收集到的您的信息都将根据法律规定的程度进行保密。在研究记录中，您

将有一个标识编号。您的个人信息在没有您的书面许可的情况下是不会公布的。但是您的记录有可能被研究主办者，伦理委员会以及相关管理机构审查。此项试验的内容有可能发表，不过您的个人信息在任何刊物上都将是保密的。

8. 费用

参加此研究不会额外增加任何费用。

9. 研究者

研究者为检验科副研究员，合作者为肿瘤科具有丰富临床经验的副主任医师，在与您交流的过程的同时，也承担健康教育者的角色，最大程度保障受试者权益。

10. 权利

参加此项临床研究是自愿的。您可以选择不参加，或者您参加后可以随时退出。您的决定不会影响您目前或将来接受的治疗或其他服务。

有关调查研究的一般问题，请随时联系本项目的研究者。

11. 受试者声明

我已经仔细阅读上述关于临床研究的内容。我的提问均已得到满意回答。此表由我自愿签署，表明我参加此项研究的愿望。签署此同意书不能免除我的合法权利。如果有疑问，或受到与研究有关的伤害，我会与我的责任护士联系。

患者签名：徐忠锋　　　　日　期：2022.1.15

联系电话：13606142888

12. 临床研究者声明

我已经仔细的向受试者介绍了以上各项的情况。因此我确保我已用我个人所学的知识向受试者清楚地解释了临床研究的性质、要求、风险以及他/她签名的合法性。任何医学、语言或者教育的障碍都不会妨碍志愿者理解这些问题。

研究者签名：周河　　　　联系电话：18862105653

日　　期：2022.1.15

如果您有与自身权益相关的任何问题，或者您想反映参与本研究过程中的不满和忧虑，请联系伦理委员会办公室，联系电话：0519-68870261/68870201。

临床研究受试者知情同意书

请您阅读以下材料，如果您愿意参加此项临床研究，您将了解此临床研究的性质以及如何参与其中。签署知情同意书将表明您已了解此临床研究并同意参加。按照我院医学伦理委员会要求在参与临床研究前签署知情同意书，这样可以保证您在了解临床研究的性质和参与的风险后再决定是否参与此临床研究。

1. 临床研究概述及目的

您被邀请参加我院的科研课题“胃癌患者肿瘤和癌旁组织中 miR-598-3p 表达”的临床调查。本研究是一项基础研究，目的是发掘促进胃癌发生发展的新指标。

2. 参加人员

（1）患者纳入标准：①术前未行任何抗肿瘤治疗及降期治疗术者；②行胃癌切除术；③术后病理诊断为胃癌患者。

（2）患者排除标准：①复发型或混合型胃癌；②术既往有恶性肿瘤病史者。

以上符合条件的患者将会参加这项研究。

3. 方法

胃癌患者肿瘤组织20例，配对的邻近非肿瘤组织20例。提取组织RNA，进行 miR-598-3p 和其他相关分子的荧光定量 PCR 检测。

4. 受益与风险

受试者可能从本项研究获得的受益：

（1）受试者将获得胃癌相关的危险因素教育；

（2）受试者可以获得与疾病相关的咨询服务。

本研究为横断面调查，仅分析病历数据，受试者承担的风险为最小风险。

5. 医疗与保护

本项临床研究的方案经过我院医学伦理委员会的审核，能很好的保护受试者的权益，并且具有科学性。参与本研究不会影响受试者的医疗方案和手段，本研究不涉及任何侵入性操作与药物试验，能很好的保护受试者的权益。

6. 自愿参加

您可以选择不参与此项临床研究，亦可以随时退出研究。

7. 保密

所有在调查中收集到的您的信息都将根据法律规定的程度进行保密。在研究记录中，您

将有一个标识编号。您的个人信息在没有您的书面许可的情况下是不会公布的。但是您的记录有可能被研究主办者，伦理委员会以及相关管理机构审查。此项试验的内容有可能发表，不过您的个人信息在任何刊物上都将是保密的。

8. 费用

参加此研究不会额外增加任何费用。

9. 研究者

研究者为检验科副研究员，合作者为肿瘤科具有丰富临床经验的副主任医师，在与您交流的过程的同时，也承担健康教育者的角色，最大程度保障受试者权益。

10. 权利

参加此项临床研究是自愿的。您可以选择不参加，或者您参加后可以随时退出。您的决定不会影响您目前或将来接受的治疗或其他服务。

有关调查研究的一般问题，请随时联系本项目的研究者。

11. 受试者声明

我已经仔细阅读上述关于临床研究的内容。我的提问均已得到满意回答。此表由我自愿签署，表明我参加此项研究的愿望。签署此同意书不能免除我的合法权利。如果有疑问，或受到与研究有关的伤害，我会与我的责任护士联系。

患者签名： 代[illegible]　　**日　期：** 2022,01,15

联系电话： 18861201088

12. 临床研究者声明

我已经仔细的向受试者介绍了以上各项的情况。因此我确保我已用我个人所学的知识向受试者清楚地解释了临床研究的性质、要求、风险以及他/她签名的合法性。任何医学、语言或者教育的障碍都不会妨碍志愿者理解这些问题。

研究者签名： 周闰　　**联系电话：** 18862105653

日　期： 2022.1.15

如果您有与自身权益相关的任何问题，或者您想反映参与本研究过程中的不满和忧虑，请联系伦理委员会办公室，联系电话：0519-68870261/68870201。

临床研究受试者知情同意书

请您阅读以下材料，如果您愿意参加此项临床研究，您将了解此临床研究的性质以及如何参与其中。签署知情同意书将表明您已了解此临床研究并同意参加。按照我院医学伦理委员会要求在参与临床研究前签署知情同意书，这样可以保证您在了解临床研究的性质和参与的风险后再决定是否参与此临床研究。

1. 临床研究概述及目的

您被邀请参加我院的科研课题“胃癌患者肿瘤和癌旁组织中 miR-598-3p 表达”的临床调查。本研究是一项基础研究，目的是发掘促进胃癌发生发展的新指标。

2. 参加人员

（1）患者纳入标准：①术前未行任何抗肿瘤治疗及降期治疗术者；②行胃癌切除术；③术后病理诊断为胃癌患者。

（2）患者排除标准：①复发型或混合型胃癌；②术既往有恶性肿瘤病史者。

以上符合条件的患者将会参加这项研究。

3. 方法

胃癌患者肿瘤组织20例，配对的邻近非肿瘤组织20例。提取组织RNA，进行 miR-598-3p 和其他相关分子的荧光定量PCR检测。

4. 受益与风险

受试者可能从本项研究获得的受益：

（1）受试者将获得胃癌相关的危险因素教育；

（2）受试者可以获得与疾病相关的咨询服务。

本研究为横断面调查，仅分析病历数据，受试者承担的风险为最小风险。

5. 医疗与保护

本项临床研究的方案经过我院医学伦理委员会的审核，能很好的保护受试者的权益，并且具有科学性。参与本研究不会影响受试者的医疗方案和手段，本研究不涉及任何侵入性操作与药物试验，能很好的保护受试者的权益。

6. 自愿参加

您可以选择不参与此项临床研究，亦可以随时退出研究。

7. 保密

所有在调查中收集到的您的信息都将根据法律规定的程度进行保密。在研究记录中，您

将有一个标识编号。您的个人信息在没有您的书面许可的情况下是不会公布的。但是您的记录有可能被研究主办者，伦理委员会以及相关管理机构审查。此项试验的内容有可能发表，不过您的个人信息在任何刊物上都将是保密的。

8. 费用

参加此研究不会额外增加任何费用。

9. 研究者

研究者为检验科副研究员，合作者为肿瘤科具有丰富临床经验的副主任医师，在与您交流的过程的同时，也承担健康教育者的角色，最大程度保障受试者权益。

10. 权利

参加此项临床研究是自愿的。您可以选择不参加，或者您参加后可以随时退出。您的决定不会影响您目前或将来接受的治疗或其他服务。

有关调查研究的一般问题，请随时联系本项目的研究者。

11. 受试者声明

我已经仔细阅读上述关于临床研究的内容。我的提问均已得到满意回答。此表由我自愿签署，表明我参加此项研究的愿望。签署此同意书不能免除我的合法权利。如果有疑问，或受到与研究有关的伤害，我会与我的责任护士联系。

患者签名：谭正荣　　　　日　期：2022.12.18

联系电话：15061126580

12. 临床研究者声明

我已经仔细的向受试者介绍了以上各项的情况。因此我确保我已用我个人所学的知识向受试者清楚地解释了临床研究的性质、要求、风险以及他/她签名的合法性。任何医学、语言或者教育的障碍都不会妨碍志愿者理解这些问题。

研究者签名：周雨　　　　联系电话：18862105653

日　　期：2022.12.18

如果您有与自身权益相关的任何问题，或者您想反映参与本研究过程中的不满和忧虑，请联系伦理委员会办公室，联系电话：0519-68870261/68870201。

临床研究受试者知情同意书

请您阅读以下材料，如果您愿意参加此项临床研究，您将了解此临床研究的性质以及如何参与其中。签署知情同意书将表明您已了解此临床研究并同意参加。按照我院医学伦理委员会要求在参与临床研究前签署知情同意书，这样可以保证您在了解临床研究的性质和参与的风险后再决定是否参与此临床研究。

1. 临床研究概述及目的

您被邀请参加我院的科研课题“胃癌患者肿瘤和癌旁组织中 miR-598-3p 表达”的临床调查。本研究是一项基础研究，目的是发掘促进胃癌发生发展的新指标。

2. 参加人员

（1）患者纳入标准：①术前未行任何抗肿瘤治疗及降期治疗术者；②行胃癌切除术；③术后病理诊断为胃癌患者。

（2）患者排除标准：①复发型或混合型胃癌；②术既往有恶性肿瘤病史者。

以上符合条件的患者将会参加这项研究。

3. 方法

胃癌患者肿瘤组织 20 例，配对的邻近非肿瘤组织 20 例。提取组织 RNA，进行 miR-598-3p 和其他相关分子的荧光定量 PCR 检测。

4. 受益与风险

受试者可能从本项研究获得的受益：

（1）受试者将获得胃癌相关的危险因素教育；

（2）受试者可以获得与疾病相关的咨询服务。

本研究为横断面调查，仅分析病历数据，受试者承担的风险为最小风险。

5. 医疗与保护

本项临床研究的方案经过我院医学伦理委员会的审核，能很好的保护受试者的权益，并且具有科学性。参与本研究不会影响受试者的医疗方案和手段，本研究不涉及任何侵入性操作与药物试验，能很好的保护受试者的权益。

6. 自愿参加

您可以选择不参与此项临床研究，亦可以随时退出研究。

7. 保密

所有在调查中收集到的您的信息都将根据法律规定的程度进行保密。在研究记录中，您

将有一个标识编号。您的个人信息在没有您的书面许可的情况下是不会公布的。但是您的记录有可能被研究主办者，伦理委员会以及相关管理机构审查。此项试验的内容有可能发表，不过您的个人信息在任何刊物上都将是保密的。

8. 费用

参加此研究不会额外增加任何费用。

9. 研究者

研究者为检验科副研究员，合作者为肿瘤科具有丰富临床经验的副主任医师，在与您交流的过程的同时，也承担健康教育者的角色，最大程度保障受试者权益。

10. 权利

参加此项临床研究是自愿的。您可以选择不参加，或者您参加后可以随时退出。您的决定不会影响您目前或将来接受的治疗或其他服务。

有关调查研究的一般问题，请随时联系本项目的研究者。

11. 受试者声明

我已经仔细阅读上述关于临床研究的内容。我的提问均已得到满意回答。此表由我自愿签署，表明我参加此项研究的愿望。签署此同意书不能免除我的合法权利。如果有疑问，或受到与研究有关的伤害，我会与我的责任护士联系。

患者签名：储小元　　　　日　期：2022年12月13日

联系电话：13915843277

12. 临床研究者声明

我已经仔细的向受试者介绍了以上各项的情况。因此我确保我已用我个人所学的知识向受试者清楚地解释了临床研究的性质、要求、风险以及他/她签名的合法性。任何医学、语言或者教育的障碍都不会妨碍志愿者理解这些问题。

研究者签名：周阳　　　　联系电话：18862105653

日　　期：2022.12.13

如果您有与自身权益相关的任何问题，或者您想反映参与本研究过程中的不满和忧虑，请联系伦理委员会办公室，联系电话：0519-68870261/68870201。

临床研究受试者知情同意书

请您阅读以下材料，如果您愿意参加此项临床研究，您将了解此临床研究的性质以及如何参与其中。签署知情同意书将表明您已了解此临床研究并同意参加。按照我院医学伦理委员会要求在参与临床研究前签署知情同意书，这样可以保证您在了解临床研究的性质和参与的风险后再决定是否参与此临床研究。

1. 临床研究概述及目的

您被邀请参加我院的科研课题“胃癌患者肿瘤和癌旁组织中 miR-598-3p 表达”的临床调查。本研究是一项基础研究，目的是发掘促进胃癌发生发展的新指标。

2. 参加人员

（1）患者纳入标准：①术前未行任何抗肿瘤治疗及降期治疗术者；②行胃癌切除术；③术后病理诊断为胃癌患者。

（2）患者排除标准：①复发型或混合型胃癌；②术既往有恶性肿瘤病史者。

以上符合条件的患者将会参加这项研究。

3. 方法

胃癌患者肿瘤组织20例，配对的邻近非肿瘤组织20例。提取组织RNA，进行miR-598-3p和其他相关分子的荧光定量PCR检测。

4. 受益与风险

受试者可能从本项研究获得的受益：

（1）受试者将获得胃癌相关的危险因素教育；

（2）受试者可以获得与疾病相关的咨询服务。

本研究为横断面调查，仅分析病历数据，受试者承担的风险为最小风险。

5. 医疗与保护

本项临床研究的方案经过我院医学伦理委员会的审核，能很好的保护受试者的权益，并且具有科学性。参与本研究不会影响受试者的医疗方案和手段，本研究不涉及任何侵入性操作与药物试验，能很好的保护受试者的权益。

6. 自愿参加

您可以选择不参与此项临床研究，亦可以随时退出研究。

7. 保密

所有在调查中收集到的您的信息都将根据法律规定的程度进行保密。在研究记录中，您

将有一个标识编号。您的个人信息在没有您的书面许可的情况下是不会公布的。但是您的记录有可能被研究主办者，伦理委员会以及相关管理机构审查。此项试验的内容有可能发表，不过您的个人信息在任何刊物上都将是保密的。

8. 费用

参加此研究不会额外增加任何费用。

9. 研究者

研究者为检验科副研究员，合作者为肿瘤科具有丰富临床经验的副主任医师，在与您交流的过程的同时，也承担健康教育者的角色，最大程度保障受试者权益。

10. 权利

参加此项临床研究是自愿的。您可以选择不参加，或者您参加后可以随时退出。您的决定不会影响您目前或将来接受的治疗或其他服务。

有关调查研究的一般问题，请随时联系本项目的研究者。

11. 受试者声明

我已经仔细阅读上述关于临床研究的内容。我的提问均已得到满意回答。此表由我自愿签署，表明我参加此项研究的愿望。签署此同意书不能免除我的合法权利。如果有疑问，或受到与研究有关的伤害，我会与我的责任护士联系。

患者签名： [illegible]　　　**日　期：** 2022年2月28日

联系电话： 15861861336

12. 临床研究者声明

我已经仔细的向受试者介绍了以上各项的情况。因此我确保我已用我个人所学的知识向受试者清楚地解释了临床研究的性质、要求、风险以及他/她签名的合法性。任何医学、语言或者教育的障碍都不会妨碍志愿者理解这些问题。

研究者签名： [illegible]　　　**联系电话：** 18862105653

日　期： 2022.2.28

如果您有与自身权益相关的任何问题，或者您想反映参与本研究过程中的不满和忧虑，请联系伦理委员会办公室，联系电话：0519-68870261/68870201。
