# Supplementary material for: Hypoxia promotes metastasis by relieving miR-598-3p-restricted glycolysis in gastric cancer
Source: J Transl Med. 2024 Mar 15;22:283. doi: 10.1186/s12967-024-04957-7 (PMC10943772; doi:10.1186/s12967-024-04957-7)
Supplement: Supplementary file 4 — Additional file 4: Additional method. [file 12967_2024_4957_MOESM4_ESM.docx]

***Cell culture, transfection, and virus***

MKN45 cells were maintained in Dulbecco’s modified Eagle’s medium (Gbico) supplemented with 10% fetal bovine serum (Gibco); SGC-7901, BGC-823 and HEK-298T cells were maintained in RPMI1640 (Gbico) supplemented with 10% fetal bovine serum. Non-hypoxic cell was cultured in 5% CO_2_, 37℃. Hypoxic cells were cultured in a modular incubator chamber (BillupsRothenberg) and supplemented with a gas mixture of 1% O_2_, 5% CO_2_, and balanced N_2_, at 37℃. Cells were transfected using Lipofectamine 3000 (Thermo Fisher Scientific). Lentiviruses, shRNA and the vectors for RMP and IGF1r knockdown were purchased from Shanghai GeneChem. Lentiviruses encoding human RMP, IGF1r, pre-miR-598-3p and Sponge were purchased from Shanghai GeneChem. Plasmids encoding RMP and IGF1r shRNA were purchased from Shanghai GeneChem. 1 μM UK5099 (Selleck, S5317, C18H12N2O2) was used to suppress pyruvate transport to mitochondrial in vitro, 1 μM R406 in vitro (Selleck, S1533, C22H23FN6O5) was used to suppress cell glycolysis in vitro.

***Analysis of miRNA Chip and Transcriptome Sequencing***

Total RNA was isolated using TRIzol reagent (Thermo Fisher Scientific). Agilent Human miRNA Microarray (8*60k) V.21.0 was used to generate miRNA expression profiles, miRNA was considered differentially expressed if its expression differed between any two samples with a fold change >2 and *p* value <0.01. Heatmap and scatter plot were generated by R package of ‘Heatmap’ and ‘Scatter plot’. Next, by using miRwalk 3.0, 53 differentially expressed microRNAs targeted genes were selected according to Context++ score percentile, targeted genes of miR-598-3p were generated by Targetsscan7.1 and miRpathDB. IGF1r has potential recognition sites for 7-8 mer seed sequences of miR-598-3p with a context++ score of -0.02, RMP has potential recognition sites for 7-1A mer seed sequences of miR-598-3p with a context++ score of -0.06 predicted by TargetScan 7.1. Next, Kyoto Encyclopedia of Genes and Genomes (KEGG) pathway enrichment analysis was performed with the ‘ClusterProfiler’ package of R and the enrichment criteria including a *p*-value < 0.05.

Transcriptome sequencing was conducted by Genewiz Co. Ltd. (Soochow, China). Gene expression levels for each transcript were estimated as the number of reads per kilobase of exon model per million mapped reads (RPKM) using only uniquely mapped reads in exonic regions. The edgeR package of R was used to analyze the difference between intergroup gene expression. The *p*-value threshold is determined by controlling the FDR (False Discovery Rate) with the Benjamini algorithm. Differentially expressed genes (DEGs) were defined as transcripts with a fold change in expression level (according to the RPKM value) ≥3.0 and a p-value < 0.05. KEGG pathway enrichment analysis was performed with the ‘ClusterProfiler’ package of R and the enrichment criteria including a *p*-value < 0.05. Heatmaps of specific genes were generated using the ‘heatmap’ package of R. Gene set enrichment analysis (GSEA) was performed using GSEA software.

***Dual-luciferase reporter assay***

Cells were grown to 70% confluence in 96-well plates, before being transfected using Lipofectamine 3000 (Thermo Fisher Scientific). psiCHECK2-RMP-3’UTR, psiCHECK2-RMP-mutant-3’UTR, psiCHECK2-IGF1r-3’UTR, psiCHECK2-IGF1r-mutant-3’UTR dual-luciferase reporter constructs were used for cotransfection. The cells were subjected to a dual-luciferase assay 24 hours after transfection. Luciferase activity was measured using the Dual-Luciferase Reporter Assay System.

***Cell invasion assay***

For the invasion assay, 5×10^4^ cells were suspended in serum-free culture medium and seeded in the top chamber of a 24-well, 8 μm, transwell unit (Corning). The unit was precoated with Matrigel (1: 8 dilution, BD Pharmingen). DMEM supplemented with 10% FBS was added to the bottom chambers of the Transwell unit, to induce metastasis. Cells were allowed to migrate for 24hr at 37℃, uninvaded cells in the top chambers were then removed and cells that had migrated into the bottom chambers were fixed, stained and quantified.

***Wound healing assay***

Cells were seeded in a fibronectin coated 6-well plate; wounds were made using 200 μl pipette tips when the cells reached 90% confluence. Cells were washed with PBS and then cultured in fresh, serum-free, culture media. The Photographs were taken post wound at 0 hours and 24 hours. The wound width was quantified using NIS-Elements software (Nikon, Japan).
