# Supplementary figures and images for: Hypoxia promotes metastasis by relieving miR-598-3p-restricted glycolysis in gastric cancer
Source: J Transl Med. 2024 Mar 15;22:283. doi: 10.1186/s12967-024-04957-7 (PMC10943772; doi:10.1186/s12967-024-04957-7)

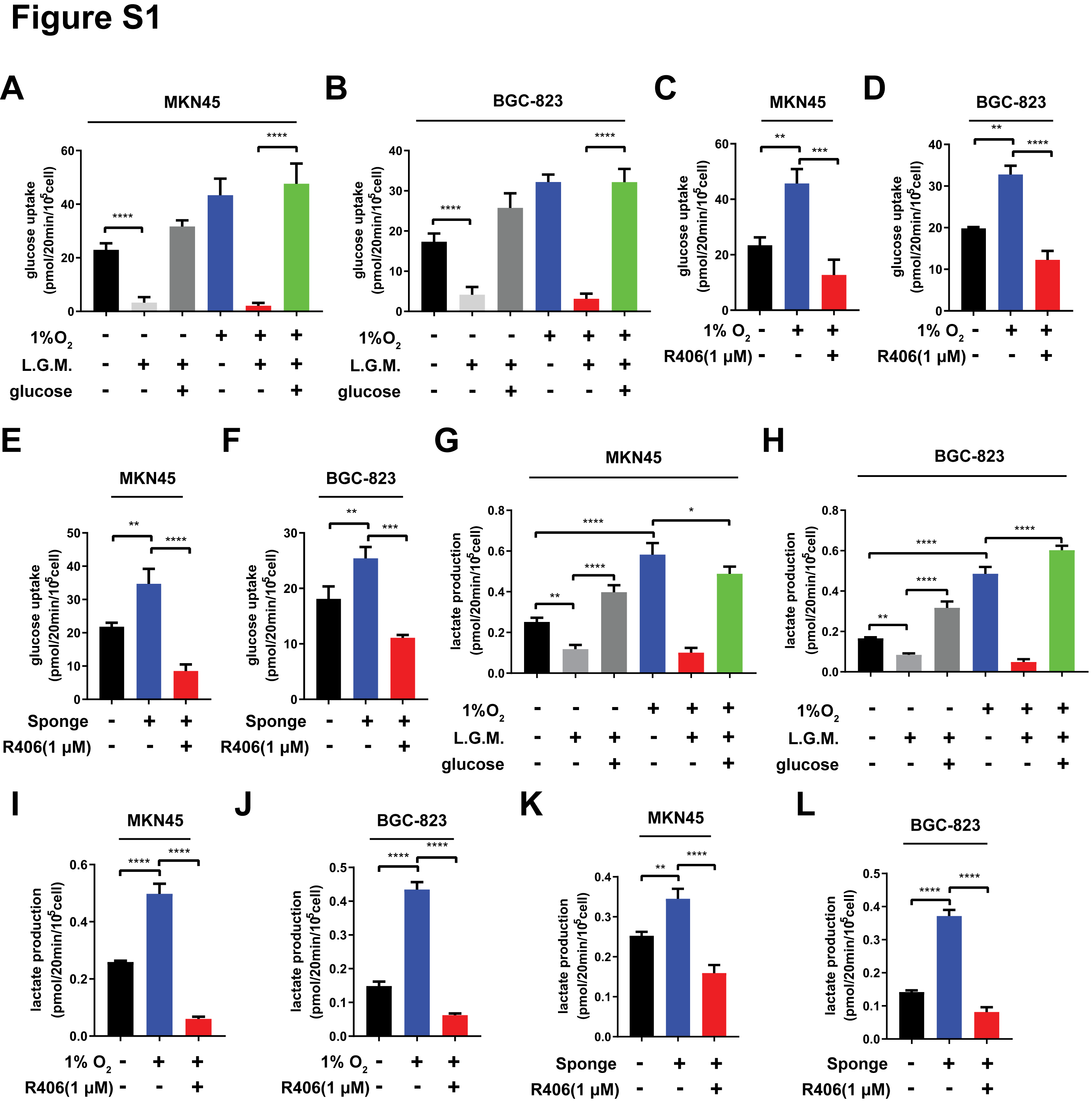

Supplement: Supplementary file 5 — Additional file 5: Figure S1. A and B. Glucose uptake of hypoxic MKN45 (A) or BGC-823 (B) cells cultured in low glucose medium (L.G.M.) 1.5 mg/L or L.G.M. supplied with glucose to 4.5 mg/L. C and D. Glucose uptake of hypoxic MKN45 (C) or BGC-823 (D) cells treated with hypoxia or 1 μM R406. E and F. MKN45 (E) or BGC-823 (F) cells transfected with Sponge or treated with 1 μM R406. G and H. Lactate production of hypoxic MKN45 (G) or BGC-823 (H) cells cultured in low glucose medium (L.G.M.) 1.5 mg/L or L.G.M. supplied with glucose to 4.5 mg/L. I and J. Lactate production of hypoxic MKN45 (I) or BGC-823 (J) cells treated with hypoxia or 1 μM R406. K and L. Lactate production of MKN45 (K) or BGC-823 (L) cells transfected with Sponge or treated with 1 μM R406. [file 12967_2024_4957_MOESM5_ESM.tif]
